# Transforming Adsorption-Energy Linear Correlations via Rescaling and Segmentation

**DOI:** 10.1021/acscatal.5c08978

**Published:** 2026-03-11

**Authors:** Nerea Azcona-Aliende, Paramaconi Rodriguez, Federico Calle-Vallejo

**Affiliations:** 1 Center of Cooperative Research on Alternative Energies (CIC energiGUNE), Basque Research and Technology Alliance, Alava Technology Park, 01510 Vitoria-Gasteiz, Spain; 2 Nano-Bio Spectroscopy Group and European Theoretical Spectroscopy Facility (ETSF), Department of Advanced Materials and Polymers: Physics, Chemistry and Technology, University of the Basque Country UPV/EHU, Avenida Tolosa 72, 20018 San Sebastián, Spain; 3 IKERBASQUE, Basque Foundation for Science, Plaza de Euskadi 5, 48009 Bilbao, Spain

**Keywords:** oxygen evolution reaction, high-throughput catalyst optimization, adsorption-energy scaling relations, rescaling, segmentation

## Abstract

Scaling relations between the adsorbed intermediates of the oxygen evolution reaction (OER) affect the efficiency of electrocatalysts. Recent efforts have been devoted to finding individual departures from scaling relations by numerous strategies. Beyond seeking particular deviations, are there any means of transforming an entire scaling relation? Here we show that the statistical nature of scaling relations is key to their harnessing. Employing a materials data set and a collection of high-throughput optimization techniques known as delta–epsilon optimization, we show the transformation of adsorption-energy correlations via rescaling and segmentation. Rescaling is a visible change in the slope and intercept of a linear relation, whereas segmentation creates two lines, one with a negative slope and another with a positive slope, the hinge point being the ideal catalyst. We illustrate these two phenomena for the scaling relations between *O, *OH, and *OOH and discuss the implications for OER electrocatalysis.

## Graphical abstract

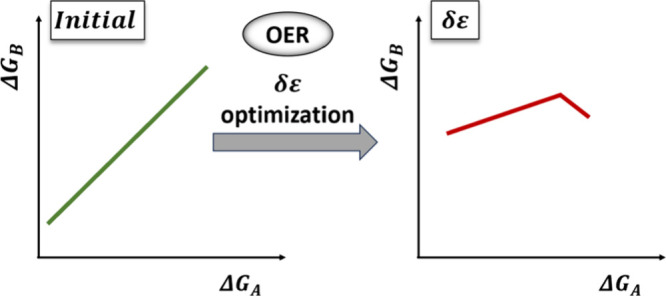

## 1 Introduction

The discovery of scaling relations two decades ago for oxygen-containing adsorbates and their subsequent extension to species containing carbon, nitrogen and sulfur species are a milestone in catalysis.^1–3^ Indeed, scaling relations revealed that adsorption energies among various materials are often interconnected: in general, simple least-squares linear regressions suffice to fit large data sets with relatively small scattering. Together with Brønsted-Evans–Polanyi relations,^4–6^ they help connect atomic-scale features of catalyst materials to their macroscopic activity.^7,8^ Although the use of scaling relations is extensive in various branches of computational catalysis and they are used to model many reactions,^9–12^ their underlying theory can be found only in a handful of works. What do we know so far and what do we not?

First things first, the scaling relation between the adsorption energies of species A and B follows the expression:^2^ Δ*G*_A_ = *m*Δ*G*_B_ + ξ. The slope (*m*) is generally positive for most adsorbates,^2^ although it may also be negative if one of the species is highly electronegative and the other is not.^13,14^ Hence, *m* depends on the chemical nature of the surface–adsorbate bond, and has been explained in terms of bond-order conservation theory.^12^ While the slope was originally rationalized in terms of the hydrogen content of the adsorbates,^2^ it was generalized to any pair of adsorbates based on the number of electrons each adsorbate requires to complete its valence shell.^13^ For instance, *OH vs *O scale with a slope of 1/2 because the O atom in *OH lacks 1 electron to fulfill the octet rule, whereas a lone O atom lacks two electrons.^2^ In turn, *S vs *O scale with a slope of 1 because both species lack 2 electrons to reach the octet.^13^ Moreover, the intercept (ξ) is linked to the specific coordination environment at the catalyst surface:^15–17^ less coordinated metal sites have more negative offsets than more coordinated sites, as long as the slope is different from 1. When the slope is 1 (or 0.75 in specific cases^16^), as is the case of *OOH vs *OH, the offset is approximately constant and does not change appreciably as a function of surface coordination.^15,18,19^

For the oxygen evolution reaction (OER, in acid: 2*H*_2_ *O* → *O*_2_ + 4*H* ^+^ + 4*e* ^–^), scaling relations are found among the three main intermediates, namely *OH, *O and *OOH.^1,15,19–22^ As said before, *OH vs *O scale with a slope of 1/2 and so do *OOH vs *O, whereas *OOH vs *OH scale with a unity slope,^15^ although covalence may introduce sizable deviations.^23^ Since the latter relation has a surface-insensitive offset, it is thought to impose thermodynamic constraints that limit the maximum efficiency of electrocatalysts, setting an intrinsic OER overpotential (η_OER_) of about 0.37 V.^18,19,24^ However, guiding principles rooted in these descriptors still lack consensus.^22,25–28^ Theoretical and experimental scientists have explored diverse design strategies: first by capitalizing on scaling relations, and in the last 15 years by breaking them, which has been regarded as pivotal for enhancing catalytic efficiency.^18–20,23,29^ Considerable effort has focused on stabilizing *OOH without concomitantly altering the adsorption energy of *OH.^19,22,30–32^ We note in passing that, to a lesser extent, attention has also been directed toward the *OH vs *O scaling relation,^21,33,34^ while recent works provide a comprehensive view on scaling relations, their manipulation and actual connection to experimental observations.^3^

As most strategies to break scaling relations are schematic or may not univocally be claimed to lead to experimental improvements of the OER activity, one may wonder whether there are quantitative catalyst optimization strategies at hand.^3,35^ Indeed, a collection of techniques called “delta-epsilon optimization” is available that quantitatively reshapes the energetic landscape of materials by means of scaling-based and scaling-free parameters, or combinations thereof.^36^ Delta-epsilon optimization predicts which OER intermediate adsorption energies need to be weakened or strengthened and by how much to reach the Sabatier activity limit. It also forecasts when scaling relations should be broken to go beyond such limit, which can be done for a specific material or in a high-throughput fashion for large materials data sets.^36,37^

An aspect of scaling relations that remains uncharted is the fact that they are statistical objects, as adsorption-energy data sets are generally well represented by the least-squares linear regressions, but still the individual data points deviate to different extents from the linear fits. Despite some examples,^20,38^ the statistical nature of scaling relations has conventionally been overlooked in the literature, so that their breaking is most often seen as a collection of unconnected cases. In this context, one may wonder whether scaling relations can be collectively modified and what the result would look like. In other words, the overall impact of numerous individual adsorption-energy changes on an overall scaling relation remains to be seen, rationalized and capitalized on.

In the light of all this, here we explore the effect of high-throughput delta-epsilon optimizations on adsorption-energy scaling relations. We go beyond the search for isolated departures from scaling relations to perform a collective analysis of catalyst optimization. Specifically, we show that their statistical nature leads to a transformation of the correlations between the adsorption energies of *O, *OH, and *OOH as the materials in them approach the zero OER overpotential limit. Within this framework we consider four optimizations corresponding to different schemes: δ optimization capitalizes on joint and proportional adsorption-energy shifts, ε optimization seizes asymmetric stabilization of a specific intermediate, δ + ε optimization sequentially combines these effects, and δε optimization simultaneously uses both parameters to provide remarkably low OER overpotentials. Importantly, high-throughput materials optimizations lead to two emerging phenomena: rescaling and segmentation. Rescaling is the drastic change of the initial slope and offset of scaling relations, whereas segmentation leads to two lines of opposing slopes and different offsets. The connecting point of the segments corresponds to the energetics of the ideal OER catalyst. In sum, we will show how the statistical nature of scaling relations opens new possibilities for their theory and practical uses in electrocatalysis.

## 2 Materials and Methods

### 2.1 Computational Framework of the OER

We employed a data set of 159 materials compiled from the literature (see Table S1), consisting of density functional theory (DFT) adsorption energies of the OER intermediates *OH, *O and *OOH (Δ*G*_OH_, Δ*G*_O_, Δ*G*_OOH_), evaluated across 11 material families. The data set includes doped and undoped TiO_2_ (35% of the data set),^39^ metalloporphyrins (23%),^23^ single-atom catalysts (SACS, 8%),^40,41^ metal monoxides (MO, 6%)^10,19,42^ and dioxides (MO_2_ including RuO_2_ and IrO_2_, 3%),^19^ perovskite oxides (SrMO_3_ (8%) and LaMO_3_ (8%),^10,19,42^ BaNiO_*x*_ (3%),^43^ Sr_*x*_Na_*y*_RuO_3_ (3%)),^44^ and double perovskite oxides (Sr_2_MIrO_6_ (3%) and La_1.5_Sr_0.5_NiMn_0.5_Ru_0.5_O_6_ (LSNMR, 2%)).^45,46^ Additional methodological details of the DFT calculations are provided in section S1 of the Supporting Information. To assess the OER activity, several reaction pathways have been proposed.^47–51^ In this work, we focused on the most widespread pathway, proposed by Rossmeisl *et al.* ^52^ and shown in eqs 1–4.

H2O+*→OH*+H++e− 1

OH*→O*+H++e− 2

O*+H2O→OOH*+H++e− 3

OOH*→*+O2+H++e− 4

In eqs 1–4, the lone asterisk designates a vacant catalytic site; when next to a species the asterisk indicates an adsorbed intermediate (*e.g.*, *O is adsorbed atomic oxygen). The free energies of eqs 1–4 are expressed as

$${\Delta G}_{1}={\Delta G}_{OH}$$ 5

$${\Delta G}_{2}={\Delta G}_{O}-{\Delta G}_{OH}$$ 6

$${\Delta G}_{3}={\Delta G}_{OOH}-{\Delta G}_{O}$$ 7

$${\Delta G}_{4}={\Delta G}_{O_{2}}-{\Delta G}_{OOH}$$ 8

where Δ*G*_O_2__ = 4.92 eV is taken from experiments at 298.15 K, as errors in the thermochemistry of O_2(g)_ prevent DFT from matching experiments.^53^ The adsorption free energies of the intermediates are referenced to water and proton–electron pairs, as shown in eq 1 for *OH. The corresponding equations for *O and *OOH appear in Section S1. While the OER has a standard equilibrium potential of *E* ^0^ = 1.23 V vs reversible hydrogen electrode (RHE), in practice it requires significant overpotentials to take place. In this context, the thermodynamic overpotential (η_OER_),^18,54^ is frequently employed as descriptor of OER activity. It is defined as the difference between the potential of the most endergonic reaction step and the equilibrium potential:

$$\eta_{OER}=\max\left({\Delta G}_{1},{\Delta G}_{2},{\Delta G}_{3},{\Delta G}_{4}\right)/e^{-}-U^{0}$$ 9

### 2.2 Delta–Epsilon Optimization

Delta–epsilon optimization is a set of quantitative strategies to optimize OER electrocatalysts by either breaking or following scaling relations.^36,37^ Considering the thermodynamic framework discussed above, we define δ, a scaling-obeying parameter, and ε, a scaling-independent parameter. Specifically, δ is a scaling-based change of Δ*G*_OH_ (δ > 0 is a weakening, δ < 0 is a strengthening) leading to a new adsorption energy: Δ*G*_OH_^’^ = Δ*G*_OH_ + δ. Because of the theoretical slopes of scaling relations, the changes for *O and *OOH are, respectively, Δ*G*_O_^’^ = Δ*G*_O_ + 2δ and Δ*G*_OOH_^’^ = Δ*G*_OOH_ + δ. Note that the coefficients of δ in these equations can be modified to reflect the actual slopes of scaling relations, which does not modify our conclusions, as shown in Section S6.

In turn, ε is a scaling-free strengthening of Δ*G*_OOH_ (hence, ε < 0) that has no effect on Δ*G*_O_ and Δ*G*_OH_ and leads to a new adsorption energy: Δ*G*_OOH_^″^ = Δ*G*_OOH_ + ε. Scaling-free optimizations are generally deemed promising to enhance the OER activity by breaking the *OOH vs *OH scaling relation.^19,55–57^ Statistically speaking, *OOH is the intermediate that deviates more strongly from the ideal OER catalyst: in our data set, the average adsorption energies of *OH, *O, and *OOH are 1.12, 2.65, and 4.31 eV, respectively, while the ideal values are 1.23, 2.46, and 3.69 eV. The individual deviations across the initial data set are such that Δ*G*_OH_ and Δ*G*_O_ deviate on average by 11 and 7%, respectively, whereas Δ*G*_OOH_ deviates by 16%. Selective modification of *OOH via hydrogen bonding, proton-acceptor functionalities, or electrolyte and interfacial field effects seems plausible, as this species is larger than *OH and has more degrees of freedom.^58–60^ Devising other scaling-free parameters is in principle possible and may help lower η_OER_. For instance, experiments have shown that selective stabilization of *OH with respect to *O in IrO_2_ via electrolyte effects enhances the OER activity by breaking the *OH vs *O scaling relation.^34^ However, such additional optimizations are not performed here, as we focus on the transformation of scaling relations, and current delta-epsilon optimizations suffice to illustrate it.

Equations 10–13 describe how variations in δ and ε affect the thermodynamics of eqs 1–4. Setting δ = ε = 0 corresponds to the initial, unoptimized energies in eqs 5–8. Besides, the sum of eqs 10–14 is again Δ*G*_O_2__.

$${\Delta G}_{1}={\Delta G}_{OH}+\delta$$ 10

$${\Delta G}_{2}={\Delta G}_{O}-{\Delta G}_{OH}+\delta$$ 11

$${\Delta G}_{3}={\Delta G}_{OOH}-{\Delta G}_{O}-\delta +\varepsilon$$ 12

$${\Delta G}_{4}={\Delta G}_{O_{2}}-{\Delta G}_{OOH}-\delta -\varepsilon$$ 13

We evaluated four approaches for our data set comprising 159 entries: scaling-based (δ) optimization, scaling-free (ε) optimization, sequential (first δ then ε, denoted δ + ε) optimization, and simultaneous (denoted δε) optimization. In general, δ can be negative or positive and is unconstrained for δ optimization. However, for δ + ε optimization, δ falls in the range of [ – 0.3 eV, 0.3 eV]. This is because δ + ε optimization is a sequential procedure in which δ is applied first. If δ is initially unrestricted, it is likely that the subsequent ε optimization yields ε = 0. Hence, – 0.3 eV < δ < 0.3 eV allows for visible scaling-based effects and leaves room for the application of ε. In turn, δε optimization is a simultaneous procedure in which both parameters are free. For each material, δ and ε were used to minimize η_OER_ using eq 9 and MS Excel Solver and the optimizations can be applied to a single material or an entire data set in a high-throughput manner.^37^

Scaling-based (δ) strategies can be experimentally implemented by *e.g.*, tuning lattice strain, coordination environment, or ligand effects, which involve proportional shifts in the adsorption energies.^43,61–66^ In turn, scaling-free (ε) optimization strategies include electrolyte effects,^34^ lateral interactions, ensemble effects and geometric effects.^3,26,67^ Sequential δ + ε optimizations emulate the experimental approach of optimizing the electrode surface via *e.g.*, strain,^43,61–63^ and then the specific active sites via tethering, hydrogen bonding, etcetera.^64–66^ A summarized workflow of δ – ε optimizations and the associated transformations of scaling relations appear in Figure S1.

## 3 Results and Discussion

### 3.1 Effect of δ and ε on Scaling Relations

In the following, the effect of δ and ε on scaling relations will be illustrated for *O and *OH, but the procedure is general and can be applied to any pair of adsorbates, including but not restricted to *O, *OH and *OOH. Since the scaling relation between *O and *OH is Δ*G*_OH_ = *m*Δ*G*_O_ + ξ, the slope and intercept can be calculated on the basis of two known adsorption energies on materials 1 and 2, as shown in eqs 14–15.

$$m=\frac{\Delta G_{OH,2}-\Delta G_{OH,1}}{\Delta G_{O,2}-\Delta G_{O,1}}=\frac{\Delta \Delta G_{OH}}{\Delta \Delta G_{O}}$$ 14

$$\xi =\Delta G_{OH,1}-m\Delta G_{O,1}=\Delta G_{OH,2}-m\Delta G_{O,2}$$ 15

Now, scaling relations result from the fit of two data sets containing numerous entries generally using the least-squares method. For Δ*G*_OH_ vs Δ*G*_O_, using Δ*G*_O_ to predict Δ*G*_OH_ leads to a certain error ϵ = Δ*G*_OH_^pred^ – Δ*G*_OH_, where Δ*G*_OH_^pred^ is the prediction of the *OH adsorption energy using that of *O and the equation of the linear fit.

Furthermore, if the adsorption energies of *OH on materials 1 and 2 are δ-optimized, such that Δ*G*_OH,1_^’^ = Δ*G*_OH,1_ + δ_1_ and Δ*G*_OH,2_^’^ = Δ*G*_OH,2_ + δ_2_, then the adsorption energies of *O on materials 1 and 2 are proportionally shifted, such that Δ*G*_O,1_^’^ = Δ*G*_O,1_ + δ_1_/*m* and Δ*G*_O,2_^’^ = Δ*G*_O,2_ + δ_2_/*m*, as discussed in the previous section. Under these circumstances, there is a new slope (m*), a new offset (ξ*), and new errors (ϵ_1_^’^ and ϵ_2_^’^) between the predictions of the linear fit and the DFT adsorption energies. The relationship between the new slope and the original one is

$$m^{*}=\frac{m}{1+\frac{\epsilon_{2}^{’}-\epsilon_{1}^{’}}{\Delta G_{OH,2}^{’}-\Delta G_{OH,1}^{’}}}=\frac{m}{1+\Delta \epsilon^{’}/\Delta {\Delta G}_{OH}^{’}}$$ 16

Equation 16 suggests that δ and ε can reshape the fundamental relation between adsorption energies because of the departures of the data points from the linear fit. The only case in which δ optimization does not change the slope is when the difference between the errors is null (Δϵ = 0), which corresponds to a line with a unity correlation coefficient or, more generally, to a line in which the materials have identical departures. Knowing the new slope (m*) and the coordinates of one of the data points, *e.g.,* (Δ*G*_O,1_^’^, Δ*G*_OH,1_^’^), one can easily calculate the offset with a modified version of eq 15 (ξ* = Δ*G*_OH,1_^’^ – *m*_δ_ · Δ*G*_O,1_^’^). A derivation illustrating how statistical deviations lead to changes in the slopes of scaling relations under δ, ε and δε optimizations, together with representative numerical examples are provided in Section S3.

An intriguing prediction of eq 16 is that negative slopes may arise, which are generally uncommon,^14^ especially among *O, *OH and *OOH. The coexistence of negative and positive slopes forms the basis of the phenomenon referred to as segmentation that will be presented and discussed in the next subsections. In particular, the slope after δ optimization will be negative in two cases: (i) when Δϵ^’^ < – ΔΔ*G*_OH_^’^, if ΔΔ*G*_OH_^’^ > 0; or (ii) when Δϵ^’^ > – ΔΔ*G*_OH_^’^, if ΔΔ*G*_OH_^’^ < 0.

Of course, the analysis leading to eq 16 can be made for the other delta-epsilon optimization strategies. Numerical examples to illustrate this with two materials, three materials and the full SAC family are shown in Section S3. With the predictions of this subsection in mind, we will now dissect the impact of high-throughput optimizations on specific scaling relations.

### 3.2 *OH vs *O

The number of electrochemical steps larger than 1.23 eV in eqs 5–8, denoted as *n*, has been shown to correlate with the OER activity.^68^ Physically, *n* reflects the number of electrochemical OER steps that are thermodynamically uphill at the equilibrium potential (1.23 V vs RHE) or, analogously, those larger than 1.23 eV when the applied potential is 0 V vs RHE.^37,68,69^ Statistically speaking, materials with *n* = 3 are highly active for the OER, whereas materials with *n* = 1,2 are neither active nor inactive per se.^70^ Accordingly, searching for *n* = 3 is a swift and convenient way of anticipating the OER activity of materials. Specifically, materials with *n* = 3 are statistically inclined to display low overpotentials, and unconstrained delta-epsilon optimizations generally result in an increase of *n* from 1 and/or 2 to 3.^37^ This is why in this manuscript the data are classified as a function of *n*. As shown in Figure 1a and Table 1, the initial slope (*m* = 0.51 ± 0.02) shows only a minor deviation from the theoretical value of 1/2.^2,15,18,19,23^

**Figure 1:**
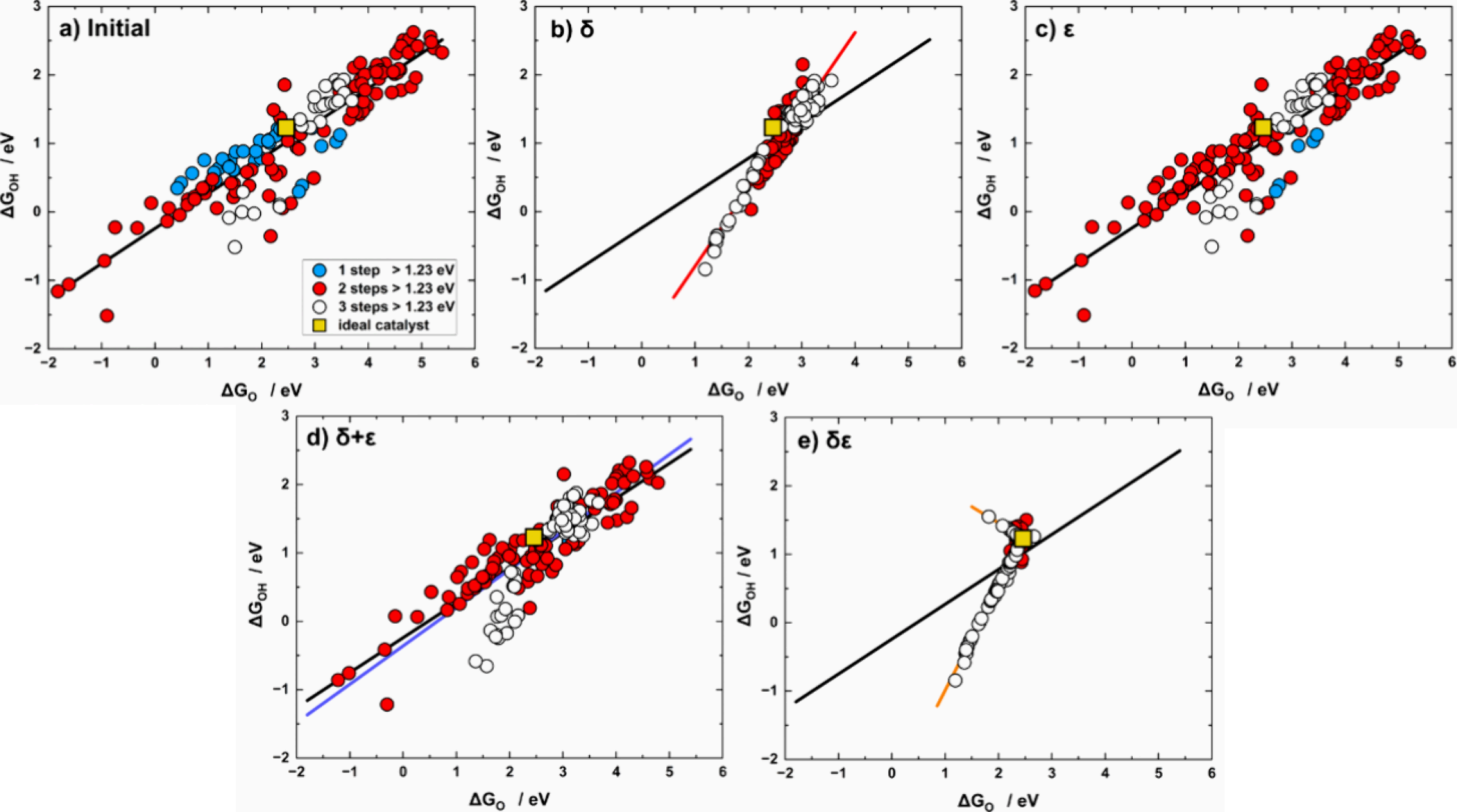
Scaling relations for Δ*G*_OH_ vs Δ*G*_O_ for (a) initial data, and data after (b) δ, (c) ε, (d) δ + ε, and (e) δε optimizations. The data are classified depending on the number of electrochemical steps larger than 1.23 eV at 0 V vs RHE: blue (*n* = 1), red (*n* = 2), and white (*n* = 3). The ideal catalyst with η_OER_ = 0 and *n* = 4 is the yellow square. Colored lines correspond to the scaling relations obtained after each optimization, while the solid black line represents the initial scaling relation in all panels.

**Table 1:** Scaling Relations of Δ*G*_OH_ vs Δ*G*_O_ (Δ*G*_OH_ = *m*Δ*G*_O_ + ξ) Derived from Initial and Optimized Data including Correlation Coefficients (*r*)­^a^

	*m*	ξ (eV)	*r*
initial	0.51 ± 0.02	–0.24 ± 0.06	0.91
δ	1.14 ± 0.03	–1.94 ± 0.08	0.95
ε	0.51 ± 0.02	–0.24 ± 0.06	0.91
δ + ε	0.56 ± 0.03	–0.36 ± 0.07	0.87
(δε)_1_	1.51 ± 0.02	–2.51 ± 0.04	0.99
(δε)_2_	–0.47 ± 0.05	2.40 ± 0.12	0.84
ideal	0.50	0.00	

a (δε)_1_ applies to Δ*G*_OH_ < 1.23 eV, while (δε)_2_ to Δ*G*_OH_ > 1.23 eV*.*

The intercept is – 0.24 ± 0.06 eV. In principle, this intercept should be zero if the slope is 1/2, as the thermodynamically ideal OER catalyst has (Δ*G*_O_^ideal^, Δ*G*_OH_^ideal^) = (2.46,1.23) eV. However, scaling relations are statistical in nature and the ideal catalyst is observed in Figure 1a to be close to the line without exactly falling over it. We note that structural and electronic heterogeneities among materials can induce deviations from the ideal linear relation. In particular, factors such as adsorption-site coordination, stoichiometry and adsorbate solvation may cause these deviations.^10,15,19,23,42^

We observe in Figure 1a,b that δ optimization shortens the ranges of Δ*G*_O_ and Δ*G*_OH_ and most sites now have *n* = 2,3. However, the most noticeable change observed is the rescaling of the linear correlation: the resulting slope and offset are *m*_δ_ = 1.14 ± 0.03 and ξ_δ_ = – 1.94 ± 0.08 eV, respectively. Hence, as predicted by eq 16, δ optimization alters both parameters. The observed increase in the slope together with the strengthening of the offset suggests an enhanced coupling between *OH and the active sites compared to *O.

Since ε affects neither Δ*G*_O_ nor Δ*G*_OH_, there are no differences with respect to the initial values upon ε optimization, see Figure 1a,c. The only noticeable difference is an increase in the number of materials with *n* = 2,3 at the expense of a decrease in those with *n* = 1. Furthermore, sequential δ + ε optimization (blue line in Figure 1d) leads to slight variations from the initial slope and intercept values, resulting from the constrained application of δ. The limited effect of δ causes the range of energies to narrow slightly relative to the initial ones. The slightly lower correlation coefficient in Table 1 (0.87 vs 0.91) stems from the fact that materials with *n* = 2 appear to follow different trends compared to those with *n* = 3. These differences between data with *n* = 2 and *n* = 3 are also apparent in Figure 1a,c. Section S4 provides a detailed analysis of this behavior.

Finally, upon simultaneous δε optimization materials are distributed in a reduced range of energies in Figure 1e and most of them display *n* = 3. This optimization induces a severe transformation of the Δ*G*_OH_ vs Δ*G*_O_ scaling relation. In fact, we observe for the first time the segmentation of the correlation: one part with a positive slope and another with a negative slope, and the hinge point is the ideal catalyst, for which (Δ*G*_O_^ideal^, Δ*G*_OH_^ideal^) = (2.46,1.23) eV. The segment with the positive slope ((δε)_1_ in Table 1) is close to that of δ optimization with a slope of 1.53, and generally corresponds to materials with steps 2, 3, and 4 above 1.23 eV. This line exhibits the highest correlation coefficient in Table 1, suggesting improved data alignment after optimization, which is characteristic of materials with *n* = 3, as shown in Table S7.

Conversely, the segment with the negative slope ((δε)_2_ in Table 1) contains materials with steps 1, 3, and 4 above 1.23 eV, and the slope is as low as – 0.34 ± 0.06 while the intercept is as high as 2.10 ± 0.15 eV. This line has the lowest correlation coefficient (*r* = 0.64), which agrees with previous observations that negative scaling relations have smaller correlation coefficients than the positive ones.^14^ The location of the data points on either segment depends on features of step 1: materials with step 1 below 1.23 eV are on the positive segment (72% of analyzed materials), while those for which step 1 is above 1.23 eV are located on the negative one (28%). Section S4 shows that a material is generally on the negative segment when Δ*G*_O, opt_ – 2Δ*G*_OH, opt_ < 0 (and Δ*G*_OH, opt_ > 1.23 eV). This condition is the same before and after all optimizations: Δ*G*_O, initial_ – 2Δ*G*_OH, initial_ = Δ*G*_O, opt_ – 2Δ*G*_OH, opt_, so one can predict the location of optimized catalysts even before optimizing them. Figure S2 shows that materials on the negative segment are above Δ*G*_OH_ = 0.5 Δ*G*_O_ in the initial scaling of Δ*G*_OH_ vs Δ*G*_O_. Finally, Section S7 shows that segmentation is independent of the magnitudes of δ and ε.

### 3.3 OOH* vs *OH

Arguably, the scaling relation of Δ*G*_OOH_ vs Δ*G*_OH_ is the most studied one to evaluate and overcome intrinsic electrocatalytic limitations.^18,19,22,24,28,31,36,69^ As shown in Figure 2 and Table 2, the adsorption energies are initially connected as Δ*G*_OOH_ = 0.82 Δ*G*_OH_ + 3.39. In theory, this scaling relation should exhibit a slope of 1, as the O atom bound to the surface in *OH and *OOH lacks an electron to reach the octet.^15,18^ Deviations to lower values are usually related to an increase in the covalent character of surface-OOH bonds.^10,23,39^ Regarding the intercept, literature values are typically around 3.2 ± 0.2 eV for metals, alloys, and oxides, provided that the slope is unity.^31,38^ Hence, our value of 3.39 ± 0.03 eV lies within the expected range. We note that this scaling relation does not include the ideal catalyst (Figure 2a): (Δ*G*_OH_^ideal^, Δ*G*_OOH_^ideal^) = (1.23 eV,3.69 eV), which supposedly is the source of experimental OER inefficiencies.^18,19^

**Figure 2:**
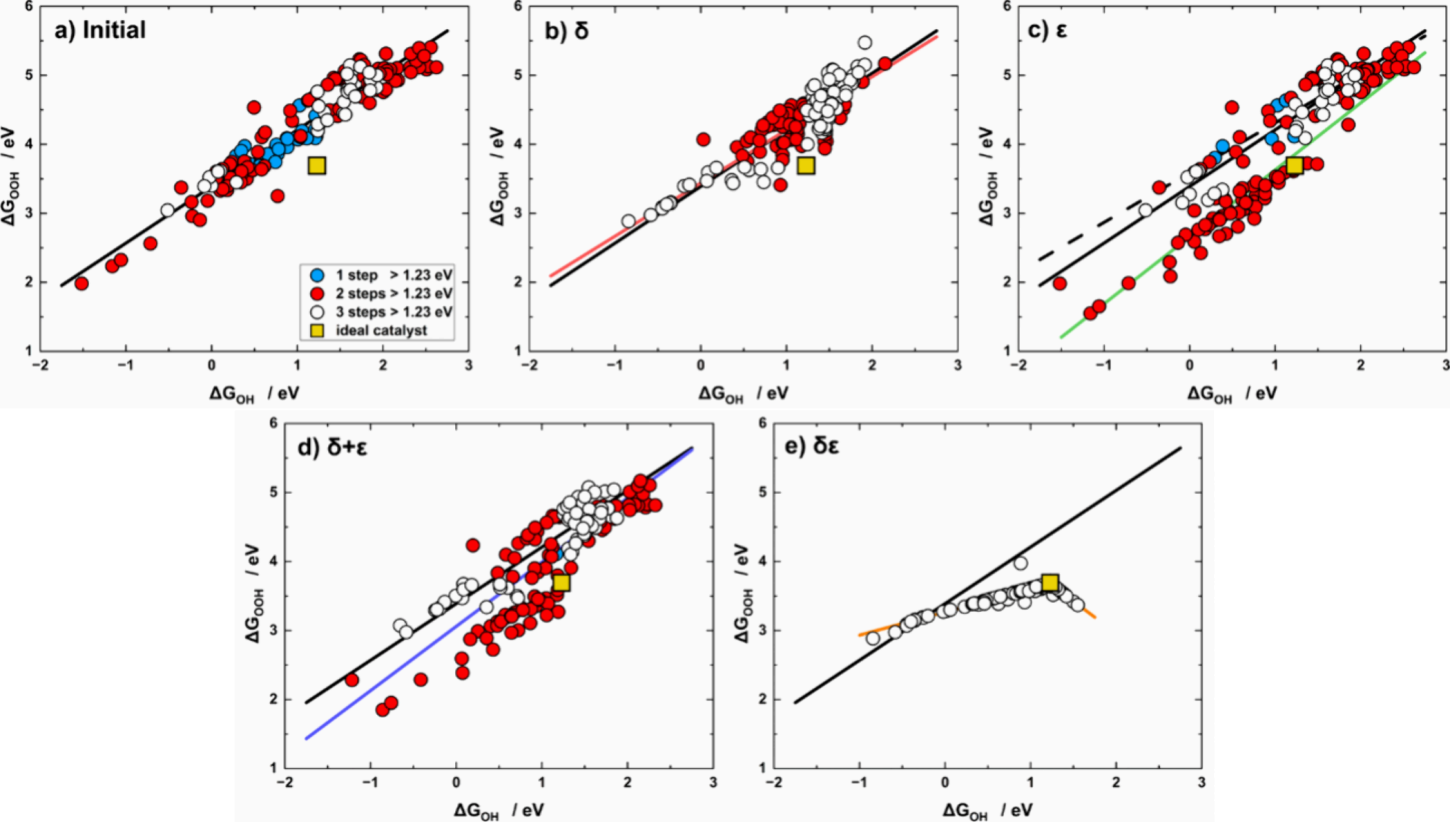
Scaling relations of Δ*G*_OOH_ vs Δ*G*_OH_ for (a) initial data, and data after (b) δ, (c) ε, (d) δ + ε, and (e) δε optimizations. The data are classified depending on the number of electrochemical steps larger than 1.23 eV at 0 V vs RHE: blue (*n* = 1), red (*n* = 2), and white (*n* = 3). The ideal catalyst with η_OER_ = 0 and *n* = 4 is the yellow square. Colored lines correspond to the scaling relations obtained after each optimization, while the solid black line represents the initial scaling relation in all panels; the dashed black line is the scaling relation for materials unaffected by ε optimization.

**Table 2:** Scaling Relations of Δ*G*_OOH_ vs Δ*G*_OH_ (Δ*G*_OOH_ = *m*Δ*G*_OH_ + ξ) Derived from Initial and Optimized Data including the Correlation Coefficient (*r*)­^a^

	*m*	ξ (eV)	*r*
initial	0.82 ± 0.02	3.39 ± 0.03	0.95
δ	0.77 ± 0.03	3.44 ± 0.04	0.88
ε (≠ 0)	0.97 ± 0.06	2.66 ± 0.05	0.89
ε (= 0)	0.72 ± 0.03	3.59 ± 0.06	0.92
δ + ε	0.93 ± 0.04	3.06 ± 0.06	0.86
(δε)_1_	0.34 ± 0.01	3.27 ± 0.01	0.94
(δε)_2_	–0.93 ± 0.04	4.82 ± 0.05	0.97
ideal	1.00	2.46	

a (δε)_1_ applies to Δ*G*_OH_ < 1.23 eV, while (δε)_2_ to Δ*G*_OH_ > 1.23 eV*.*

As shown in Figure 2b, δ optimization does not cause large deviations from the initial slope (0.77 vs 0.82) and intercept (3.44 vs 3.39 eV), which suggests that the scaling between *OOH and *OH is more difficult to transform compared to that of *O and *OH. However, more materials with *n* = 3 and a clustering of data points around the ideal value (Δ*G*_OH_^ideal^ = 1.23 eV) can be seen in Figure 2b.

Figure 2c illustrates the effect of ε optimization on Δ*G*_OOH_, where the green line corresponds to the cases where ε ≠ 0 and the black dashed line represents materials unaffected by ε optimization (ε = 0). Materials for which ε ≠ 0 (green line) exhibit a slightly higher slope (0.97 ± 0.06) and a lower intercept (2.66 ± 0.05 eV), approaching the ideal scaling line (Δ*G*_OOH_ = Δ*G*_OH_ + 2.46) and including the ideal OER catalyst. Meanwhile, materials for which ε = 0 (black dashed line in Figure 2c) are those for which the highest-energy step is not step 3. For those materials the slope marginally decreases (0.72 ± 0.03) and the intercept increases (3.59 ± 0.06 eV), thereby deviating further from the ideal scaling line.

δ + ε optimization in Figure 2d (blue line) shows similar effects as the optimization with ε ≠ 0 in Figure 2c, plus a slight decrease in the data ranges due to the application of δ. Note that δ + ε optimization is fit by a single line (blue), as all materials were optimized with δ, while ε was applied only when feasible, as done in Figure 1d.

Figure 2e shows that simultaneous δε optimization leads to a segmentation of Δ*G*_OOH_ vs Δ*G*_OH_ similar to that of Δ*G*_OH_ vs Δ*G*_O_. Two distinct regimes emerge again: for Δ*G*_OH_ < 1.23 eV, the segment has a positive slope of 0.35 ± 0.01 and an intercept of 3.26 ± 0.01 eV ((δε)_1_ in Table 2). This segment encompasses the majority of materials, most of which exhibit steps 2, 3, and 4 above 1.23 eV. Conversely, for Δ*G*_OH_ > 1.23 eV, the segment has a negative slope of −0.94 ± 0.06 and the offset is as large as 4.83 ± 0.07 eV ((δε)_2_ in Table 2), where materials with steps 1, 3, and 4 above 1.23 eV are predominantly found. Interestingly, both segments include the ideal catalyst and deviate considerably from the initial trend in Figure 2a.^26,58,67^ The distribution of materials across the segments in Figure 2e resembles the one in Figure 1e. Again, the placement of the data points along the two segments is dictated by step 1: catalysts with step 1 below 1.23 eV fall on the left segment (72%), whereas those with step 1 above 1.23 eV are on the right (28%).

In sum, we have shown in Figure 2 that it is possible to systematically transform the entire *OOH vs *OH scaling relation. The rescaling and segmentation in Figure 2 differ from most works in the literature, where the efforts are most often aimed at finding a few specific outliers and no trends emerge upon the breaking of the initial relation.^26,32,35,58,67,71^

### 3.4 *OOH vs *O

We analyze the scaling relation of Δ*G*_OOH_ vs Δ*G*_O_ in Figure 3 and Table 3. The initial slope (0.45 ± 0.01) is close to the expected value of 1/2, whereas the intercept amounts to 3.11 ± 0.04 eV, which is visibly different from the ideal one (2.46 eV). As a result, the ideal catalyst is relatively far from the linear fit but some scattered data points are around it. Again, after δ optimization (Figure 3b), the linear correlation is rescaled: the slope increases by nearly a factor of 2 (1.00 ± 0.02), while the intercept (1.61 ± 0.07 eV) is reduced by nearly one-half of the initial one (3.11 ± 0.04 eV). As a result of the rescaling, the ideal catalyst becomes part of the trend in Figure 3b.

**Figure 3:**
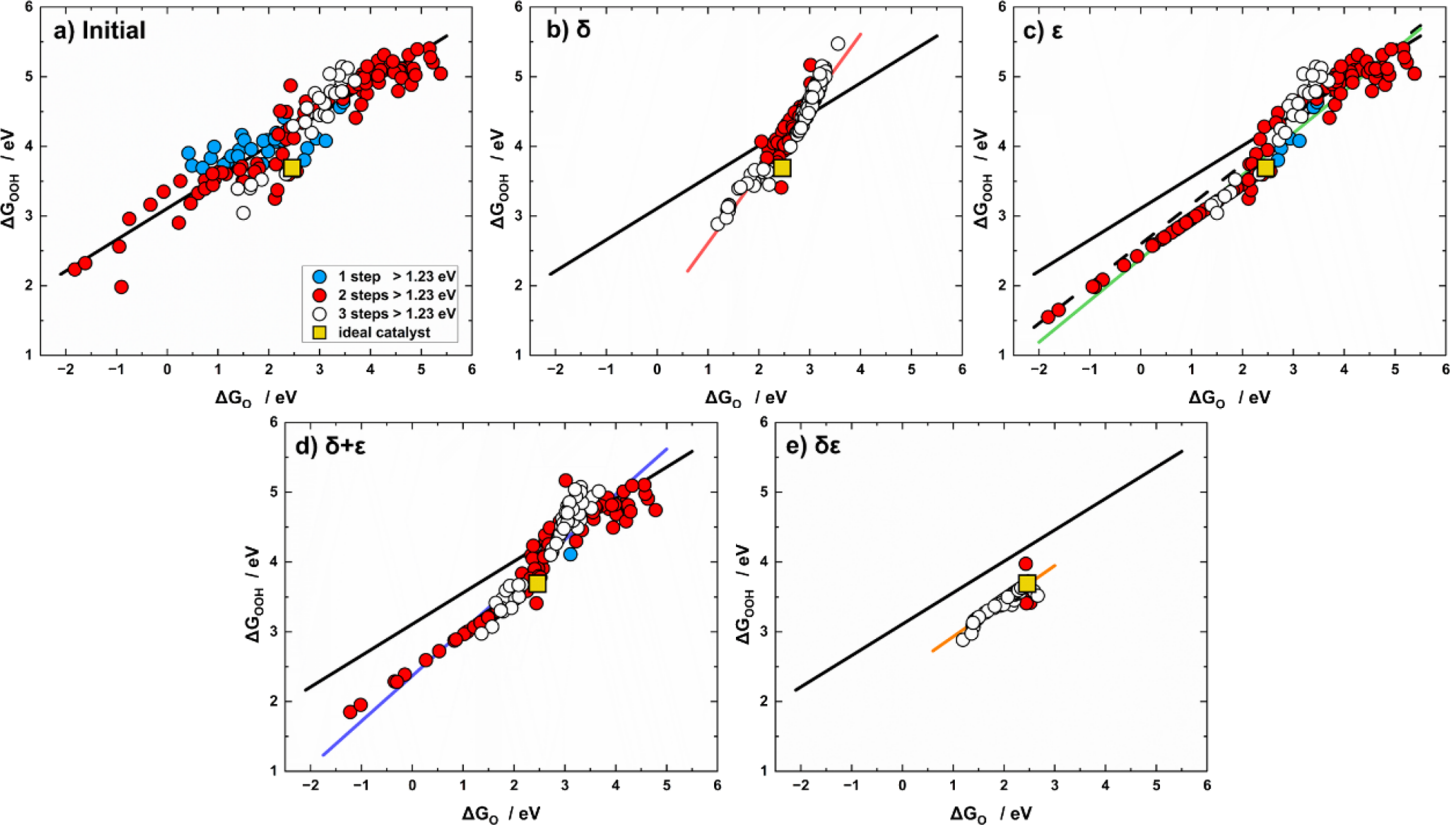
Scaling relations of Δ*G*_OOH_ vs Δ*G*_O_ for (a) initial data, and data after (b) δ, (c) ε, (d) δ + ε, and (e) δε optimizations. The data are classified depending on the number of electrochemical steps above 1.23 eV at 0 V vs RHE: blue (*n* = 1), red (*n* = 2), and white (*n* = 3). The ideal catalyst with η_OER_ = 0 and *n* = 4 is in yellow. Colored lines correspond to the scaling relations obtained after each optimization, while the solid black line represents the initial scaling relation in all panels; the dashed black line is the scaling relation for materials unaffected by ε optimization.

**Table 3:** Scaling Relations of Δ*G*_OOH_ vs Δ*G*_O_ (Δ*G*_OOH_ = *m*Δ*G*_O_ + ξ) Derived from Initial and Optimized Data including the Correlation Coefficient (*r*)

	*m*	ξ (eV)	*r*
initial	0.45 ± 0.01	3.11 ± 0.04	0.93
δ	1.00 ± 0.02	1.61 ± 0.07	0.96
ε (≠ 0)	0.60 ± 0.02	2.39 ± 0.03	0.96
ε (= 0)	0.57 ± 0.03	2.60 ± 0.10	0.91
δ + ε	0.65 ± 0.02	2.37 ± 0.05	0.95
δε	0.51 ± 0.02	2.42 ± 0.03	0.93
ideal	0.50	2.46	

The ε-optimized data set in Figure 3c is less scattered than the initial one. As in Figure 2c, the green and black dashed lines represent the correlations for materials with and without ε optimization, respectively. When ε ≠ 0 (green line), the slope increases to 0.60 ± 0.02 and the intercept is slightly reduced (2.39 ± 0.03 eV), which are both close to the ideal catalyst, see Table 3. The same effect is observed for materials with ε = 0 (black dashed line), which indicates that materials with ε ≠ 0 display the largest deviations from ideality in the initial data set. Moreover, the results of δ + ε optimization (blue line in Figure 3d) are similar to those obtained with ε optimization, which is consistently observed for the other scaling relations in Tables 1–2. This scaling relation also shows a visible dependence on *n*, as reflected in Table S8.

As shown in Figure 3e, simultaneous δε optimization again results in a significant alteration of the scaling relations. It is interesting to note that in this case nearly all materials cluster around the ideal value and we do not observe any apparent segmentation. This was expected, as the hinge point of the segmented scaling relations in Figures 1e and 2e is in both cases Δ*G*_OH_ = 1.23 eV and the trends are proportional, such that upon combining the segmented trends a single linear relation should be found.^13^

To illustrate this, we note that the line in Figure 3e and Table 3 can be derived from the four individual segments combined pairwise: two segments corresponding to *OOH vs *OH and *OH vs *O on materials Δ*G*_OH_ < 1.23 eV, and two segments with Δ*G*_OH_ > 1.23 eV, denoted as (δε)_1_ and (δε)_2_ in Tables 1 and 2, respectively. As summarized in Table 4, the first combination yields a linear trend (α) containing 72% of the data, whereas the second combination yields another line (β) with 28% of the data. The line obtained from these two weighted contributions closely matches the scaling relation in Table 3. In Table 4 the δε optimized slope is close to the weighted and ideal values (0.51 vs 0.48 and 0.50), which holds true for the offset (2.42 vs 2.50 and 2.46 eV). Further details are provided in Section S5.

**Table 4:** Scaling Relations of Δ*G*_OOH_ vs Δ*G*_O_ (Δ*G*_OOH_ = *m*Δ*G*_O_ + ξ) Derived from δε Optimized Data^a^

segment	*m*	ξ (eV)	weight (%)
α	0.54 ± 0.02	2.36 ± 0.03	72
β	0.32 ± 0.06	2.86 ± 0.20	28
0.72α + 0.28β	0.48 ± 0.02	2.50 ± 0.06	100
δε	0.51 ± 0.02	2.42 ± 0.03	100

a The weights correspond to the percentage of data points belonging to the segments.

Finally, in view of the similarity between the ideal and optimized slopes and intercepts, the ideal catalyst is part of the trend in Figure 3e. We note that while the majority of materials are close to the ideal catalyst, materials with steps 2, 3, and 4 above 1.23 eV exhibit larger departures from the trend.

### 3.5 OER Electrocatalysis: Breaking vs Transforming Scaling Relations

Deviations from the ideal *OOH vs *OH energy separation are thought to underlie the inefficiency of OER electrocatalysts.^3,18,19,22,24,28,31,36,69^ In fact, it is widely believed that materials approaching the optimal 2.46 eV difference between Δ*G*_OOH_ and Δ*G*_OH_ (see Table 2) are expected to exhibit low OER thermodynamic overpotentials (η_OER_), whereas larger deviations should correspond to less active materials. The parameter γ_OOH/OH_ was defined to quantify the degree of breaking of the *OOH vs *OH scaling relation:^22^

$$\gamma_{OOH/OH}=\left(\Delta G_{OOH}-\Delta G_{OH}-2.46\right)/2e^{-}$$ 17

The ideal catalyst has γ_OOH/OH_ = 0, while most materials have 0 < γ_OOH/OH_ < 1. As shown in Figure 4a, in the initial data set, η_OER_ does not show an apparent correlation with γ_OOH/OH_. In fact, some materials close to the optimum value exhibit large overpotentials, while others with larger γ_OOH/OH_ perform better. In general, there is a gap of nearly 0.37 V between the most active catalysts and the ideal one, which we will hereafter refer to as the “intrinsic overpotential”.^18^ Furthermore, δ optimization leads to a clearer correlation between γ_OOH/OH_ and η_OER_ in Figure 4b, characterized by a slope close to unity (0.82 ± 0.05) and an intercept close to zero (0.11 ± 0.02 eV), see Table 5. Some materials with *n* = 2 depart from the linear trend consisting mainly of materials with *n* = 3, causing the observed variations in slope and intercept, and a lower correlation coefficient (0.79). In addition, the intrinsic overpotential is visibly shortened upon δ optimization.

**Figure 4:**
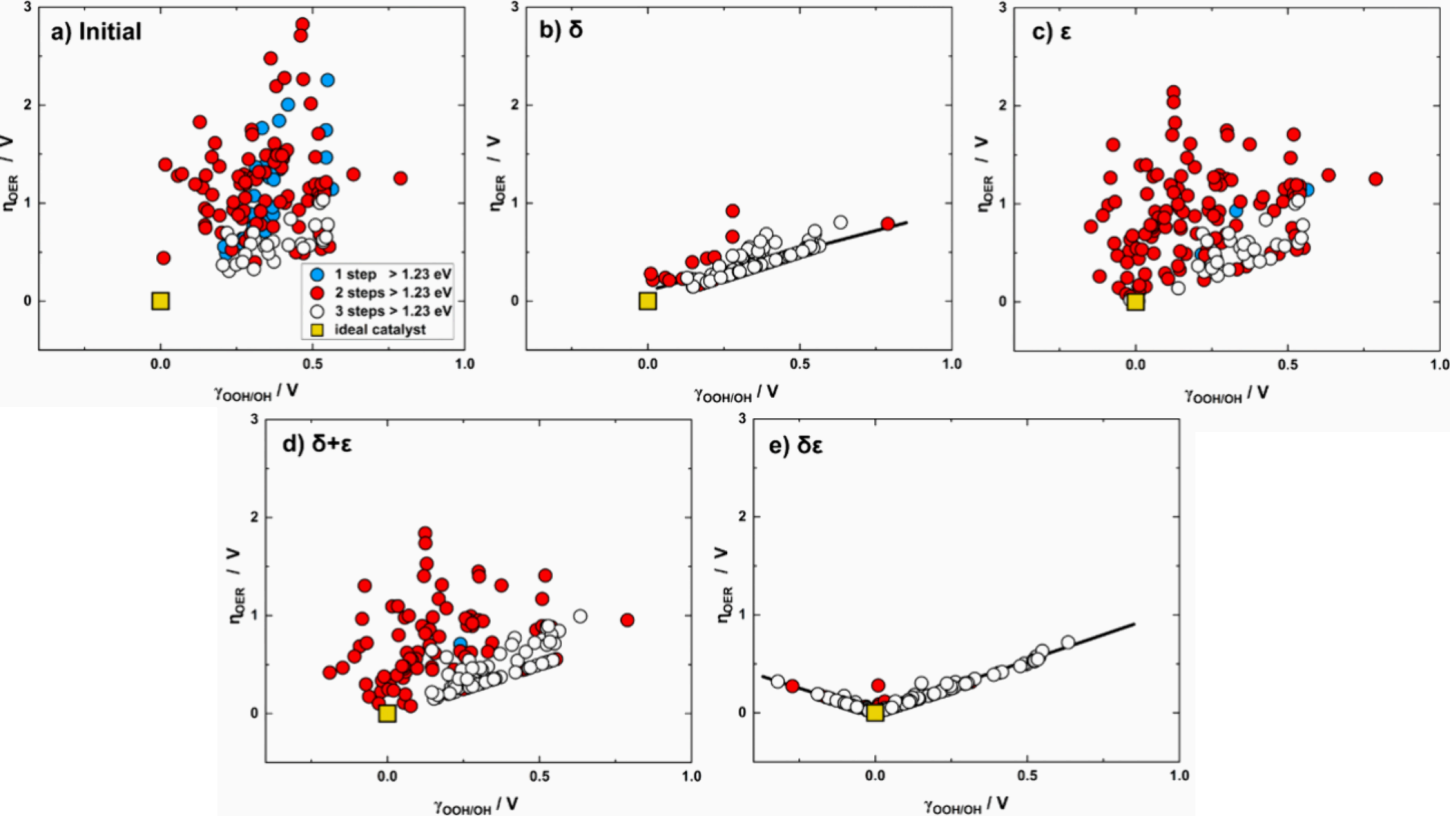
Calculated OER overpotentials (η_OER_) as a function of their departures from the ideal *OOH vs *OH energetic separation of 2.46 eV for (a) initial data, and data upon (b) δ, (c) ε, (d) δ + ε, and (e) δε optimizations. Data are classified depending on the number of electrochemical steps above 1.23 eV at 0 V vs RHE: blue (*n* = 1), red (*n* = 2), and white (*n* = 3). Linear fits are provided in Table 5.

**Table 5:** Overpotential as a Function of the Deviation from the Ideal *OOH vs *OH Energy Difference (η_OER_ = a γ_OOH/OH_ + *b*) for δ and δε Optimized Data Sets including the Correlation Coefficient (*r*)

	*a*	*b* (V)	*r*
δ	0.82 ± 0.05	0.11 ± 0.02	0.79
(δε)_1_	1.06 ± 0.02	0.01 ± 0.01	0.97
(δε)_2_	–0.99 ± 0.03	0.01 ± 0.01	0.98

Figure 4c illustrates the effect of ε optimization, which was shown in Figure 2 to disrupt the scaling relation between *OOH and *OH. Although an overall correlation between γ_OOH/OH_ and η_OER_ is less evident compared to δ optimization, a tangible lowering of the intrinsic overpotential is observed, with certain materials being remarkably close to the ideal catalyst. What is more, ε optimization results in materials exhibiting γ_OOH/OH_ < 0, which has not been reported previously in the literature. These negative values reflect the fact that low overpotentials can also be found when Δ*G*_OOH_ – Δ*G*_OH_ < 2.46 eV. In the case of δ + ε sequential optimization, the effect is similar to that observed for ε optimization, see Figures 4c-d. Nevertheless, the combination of δ and ε yields lower average η_OER_ and γ_OOH/OH_ across the data set, and a linear correlation for data with *n* = 3 can be seen.

Finally, simultaneous δε optimization leads in Figure 4e to a full reorganization of the materials in an inverted volcano distribution. The apex of this volcano corresponds to the ideal catalyst with (γ_OOH/OH_, η_OER_) = (0,0). On both sides of the inverted volcano, the linear trends display slopes of ± 1 and intercepts close to zero (see Table 5). Note that the two segments in Figure 4e correspond to those in Figure 2e (see Section S4).

The materials in Figure 4e generally display *n* = 3 and most data points are located on the right side, where steps 2, 3, and 4 are generally above 1.23 eV. Conversely, on the left side of the inverted volcano, steps 1, 3, and 4 are typically above 1.23 eV. The negative values of γ_OOH/OH_ in Figure 4 show that 2.46 eV is the ideal energetic difference but not the lowest allowed by thermodynamics, such that low OER overpotentials can also be obtained from materials with Δ*G*_OOH_ – Δ*G*_OH_ < 2.46 eV. The remaining relations (*OOH vs *O and *OH vs *O) are analyzed in Section S6. We note that ε is able to induce negative values of γ_OOH/OH_ in Figure 4c-d, but the full development of the inverted volcano takes place only in Figure 4e when δ is unconstrained.

## 4 Conclusions

The theory and practice of adsorption-energy scaling relations are paramount in catalysis. While countless works use scaling relations, their underlying theory remains underexplored, which limits our ability to harness them. For 15 years most works have been devoted to finding individual departures from scaling relations to enhance various electrocatalytic reactions. As schematized in a previous work,^3^ breaking scaling relations is only one among numerous ways of transforming these correlations. This work reports the quantitative transformation of scaling relations among the key OER intermediates (*O, *OH, *OOH) upon high-throughput catalyst optimizations. The emerging scaling relations include in all cases the ideal OER catalyst and lead to low OER overpotentials. Two remarkable phenomena emerged: rescaling and segmentation.

Rescaling is a drastic modification of the slope and intercept of scaling relations, while segmentation is their splitting into two lines with positive and negative slopes. The point connecting the segments is in all cases the ideal catalyst. These two phenomena allow materials to collectively approach the ideal OER catalyst. Interestingly, a simple error analysis shows that the two phenomena stem from the statistical nature of scaling relations. Specifically, the fact that a least-squares linear fit makes the sum of the errors equal to zero but each data point has a certain distance to the line, enables rescaling and segmentation. In particular, we observed rescaling upon δ, ε and δ + ε optimizations, while segmentation occurred after simultaneous δε optimization.

Furthermore, it is possible to link these observations to features of the OER reaction pathway: when step 1 is over 1.23 eV, larger deviations from ideal correlations are observed in scaling relations. In this regime, steps 3 and 4 are also above 1.23 eV. Conversely, if step 1 is below 1.23 eV, steps 2, 3, and 4 dominate the pathway. Hence, step 1 can be used to anticipate the reshaping of scaling relations.

Finally, it is often claimed that breaking the scaling relation between *OOH and *OH should lead to an OER activity enhancement. While this is not visible in the unoptimized data, clearer correlations develop upon delta-epsilon optimizations. Importantly, simultaneous δε optimizations lead to an inverted volcano relation between the OER overpotential and the breaking of the *OOH vs *OH scaling relation that illustrates a previously unreported phenomenon: materials with Δ*G*_OOH_ – Δ*G*_OH_ < 2.46 eV are possible and may display enhanced OER activities.

All in all, we offered here a different perspective on electrocatalyst optimization compared to the state of the art by showing the theoretical possibility to tune entire scaling relations in two ways by capitalizing on their statistical nature.

## Supplementary Material



## Notes

Details of the DFT data, data for δ – ε optimizations, transformation of scaling relations upon δ – ε optimization and their origin, dependence of the slopes on *n*, δ optimization using least-squares and theoretical slopes, Δ*G*_OOH_ vs Δ*G*_O_ upon δε optimization excluding large values of δ and ε, analysis of γ_O/OH_ and γ_OOH/O_ (PDF)
