# Supplementary material for: Transforming Adsorption-Energy Linear Correlations via Rescaling and Segmentation
Source: ACS Catal. 2026 Mar 11;16(6):5805–15. doi: 10.1021/acscatal.5c08978 (PMC13010250; doi:10.1021/acscatal.5c08978)
Supplement: Supplementary file 1 [file cs5c08978_si_001.pdf]

Supplementary Information

Transforming adsorption-energy linear correlations via rescaling and segmentation

Nerea Azcona-Aliende,[1,2] Paramaconi Rodriguez,[1,3,*] Federico Calle-Vallejo[2,3,*]

[1] *Center of Cooperative Research on Alternative Energies (CICenergiGUNE), Basque Research and Technology Alliance, Alava Technology Park, 01510 Vitoria-Gasteiz, Spain.*

[2] *Nano-Bio Spectroscopy Group and European Theoretical Spectroscopy Facility (ETSF), Department of Advanced Materials and Polymers: Physics, Chemistry and Technology, University of the Basque Country UPV/EHU, Avenida Tolosa 72, 20018 San Sebastián, Spain.*

[3] *IKERBASQUE, Basque Foundation for Science, Plaza de Euskadi 5, 48009 Bilbao, Spain.*

*Corresponding authors: PR (prodriguez@cicenergigune.com) and FCV (federico.calle@ehu.es)

Table of Contents

S1. Details of the DFT data S2

S2. Data for $\delta - \varepsilon$ optimizations S3

S3. Transformation of scaling relations upon $\delta - \varepsilon$ optimization S20

S4. Origin of the transformations after delta-epsilon optimization S22

S5. Dependence of the slopes on n S25

S6. δ optimization using least-squares and theoretical slopes S26

S7. ΔG_{OOH} *vs.* ΔG_{O} upon $\delta\varepsilon$ optimization excluding large values of δ and ε S28

S8. Analysis of $\gamma_{O/OH}$ and $\gamma_{OOH/O}$ S31

References S34

S1. Details of the DFT data

As mentioned in the main text, we collected the data from the literature (see section S2). All materials were optimized using density functional theory (DFT) calculations, generally with the RPBE exchange-correlation functional. We note that as adsorption energies depend on the exchange-correlation functional, the use of a different functional (e.g., PBE) would lead to different absolute adsorption energies, and consequently to different values of δ and ε. However, the present analysis does not aim at providing functional-specific optimization parameters, but rather at examining how adsorption scaling relations are rescaled and segmented under different optimization schemes.

In general, the calculations were spin-unrestricted when necessary, and the convergence criterion for the ionic loops was that the maximum force was to be below 0.05 eV/Å. The computational hydrogen electrode (CHE) approach was employed to assess the energetics of proton-electron pairs.[1] Accordingly, in Equations 1-4 in the main text, the energetics of $H_2(g)$ is used instead of $2(H^+ + e^-)$, since these species are in equilibrium under the conditions defined by the CHE. The adsorption energies of *O, *OH and *OOH were also computed using this approach, following the reactions below. Note that equation S2 is equivalent to equation 1 in the main text.

$$H_2O + * \rightarrow {}^*O + 2H^+ + 2e^- \quad \text{(S1)}$$

$$H_2O + * \rightarrow {}^*OH + H^+ + e^- \quad \text{(S2)}$$

$$2H_2O + * \rightarrow {}^*OOH + 3H^+ + 3e^- \quad \text{(S3)}$$

S2. Data for $\delta - \varepsilon$ optimizations

The data set compiled from the literature encompasses 159 materials from 11 different material families, as outlined in the main text. Numerous data entries were taken from studies showing close correspondence between DFT predictions and experiments. Tables S1–S5 list the initial, δ-optimized, ε-optimized, $\delta + \varepsilon$ optimized, and $\delta\varepsilon$ optimized data, while Figure S1 condenses the corresponding transformations of scaling relations. For each catalyst the tables include the adsorption energies, overpotential (η_{OER}), $\gamma_{OOH/OH}$, $\gamma_{OH/O}$ and $\gamma_{OOH/O}$.

The assessment of $\gamma_{OOH/OH}$, $\gamma_{OH/O}$ and $\gamma_{OOH/O}$ are exemplified by the initial data of two perovskite oxides: $SrCoO_3$ and $LaTiO_3$.

- For $SrCoO_3$ the adsorption energies are $\Delta G_O = 3.00\ eV,\ \ \Delta G_{OH} = 1.54\ eV$ and $\Delta G_{OOH} = 4.45\ eV$. Thus, $\gamma_{OOH/OH} = (\Delta G_{OOH} - \Delta G_{OH} - 2.46)/2e^- = (4.45 - 1.54 - 2.46)/2e^- = 0.22\ V$, $\gamma_{O/OH} = (\Delta G_O - \Delta G_{OH} - 1.23)/1e^- = (3.00 - 1.54 - 1.23)/1e^- = 0.23$ V and $\gamma_{OOH/O} = (\Delta G_{OOH} - \Delta G_O - 1.23)/1e^- = (4.45 - 3.00 - 1.23)/1e^- = 0.22\ V$.
- For $LaTiO_3$ the adsorption energies are $\Delta G_O = -1.82\ eV,\ \ \Delta G_{OH} = -1.16\ eV$ and $\Delta G_{OOH} = 2.23\ eV$. Therefore, $\gamma_{OOH/OH} = (\Delta G_{OOH} - \Delta G_{OH} - 2.46)/2e^- = (2.23 - (-1.16) - 2.46)/2e^- = 0.47\ V$, $\gamma_{O/OH} = (\Delta G_O - \Delta G_{OH} - 1.23)/1e^- = ((-1.82) - (-1.16) - 1.23)/1e^- = -1.89$ V and $\gamma_{OOH/O} = (\Delta G_{OOH} - \Delta G_O - 1.23)/1e^- = (2.23 - (-1.82) - 1.23)/1e^- = 2.82\ V$.

Table S1. Initial values of ΔG_O ,ΔG_{OH}, ΔG_{OOH} (in eV), η_{OER} (in V), $\gamma_{OOH/OH}$, $\gamma_{O/OH}$ and $\gamma_{OOH/O}$ (in V) classified according to the number of electrochemical steps over 1.23 eV (n).

a) Materials with $n = 1$

Material	Family	ΔG_O	ΔG_{OH}	ΔG_{OOH}	η_{OER}	$\gamma_{OOH/OH}$	$\gamma_{O/OH}$	$\gamma_{OOH/O}$
V-TiO_2 (6cM)	u/d TiO_2	2.76	0.39	3.97	1.14	0.56	1.14	-0.01
V-TiO_2 (6cM)	u/d TiO_2	3.40	1.03	4.56	1.14	0.54	1.14	-0.06
V-TiO_2 (6cM)	u/d TiO_2	3.47	1.13	4.63	1.11	0.52	1.11	-0.06
Nb-TiO_2 (6cM)	u/d TiO_2	2.70	0.30	3.80	1.17	0.52	1.17	-0.12
Co-FGM	SACs	2.94	1.22	4.11	0.49	0.22	0.49	-0.05
LSNMR@Mn	LSNMR	3.12	0.96	4.08	0.93	0.33	0.93	-0.27
Mo-TiO_2 (5cM)	u/d TiO_2	0.49	0.43	3.72	2.01	0.42	-1.17	2.01
Mo-TiO_2 (5cM)	u/d TiO_2	1.47	0.62	4.16	1.47	0.54	-0.38	1.47
Mo-TiO_2 (6cM)	u/d TiO_2	0.86	0.29	3.83	1.75	0.54	-0.66	1.75
W-TiO_2 (5cM)	u/d TiO_2	0.42	0.35	3.90	2.26	0.55	-1.16	2.26
Ru-TiO_2 (5cM)	u/d TiO_2	2.15	1.06	4.19	0.82	0.34	-0.14	0.82
Mn-F	Porphyrins	1.13	0.46	3.71	1.35	0.39	-0.56	1.35
Mn-BH_2	Porphyrins	1.42	0.75	3.94	1.30	0.37	-0.56	1.30
Fe-H	Porphyrins	1.19	0.63	3.77	1.36	0.34	-0.67	1.36
Fe-F	Porphyrins	1.37	0.68	3.86	1.26	0.36	-0.54	1.26
Fe-CH_3	Porphyrins	1.11	0.57	3.73	1.39	0.35	-0.70	1.39
Fe-BH_2	Porphyrins	1.51	0.88	4.09	1.35	0.38	-0.59	1.35
Co-H	Porphyrins	2.30	1.21	4.09	0.56	0.21	-0.14	0.56
Co-F	Porphyrins	1.97	1.04	4.09	0.89	0.29	-0.30	0.89
Co-OH	Porphyrins	1.65	0.88	3.96	1.07	0.31	-0.46	1.07
Co-CH_3	Porphyrins	2.15	1.01	4.07	0.69	0.30	-0.09	0.69
Co-NH_2	Porphyrins	1.26	0.77	3.86	1.37	0.31	-0.74	1.37
$SrCrO_3$	$SrMO_3$	0.92	0.76	3.99	1.84	0.39	-1.06	1.84
$SrMnO_3$	$SrMO_3$	2.30	1.21	4.41	0.88	0.37	-0.14	0.88
$SrRuO_3$	$SrMO_3$	2.26	1.11	4.09	0.59	0.26	-0.08	0.59
$LaCrO_3$	$LaMO_3$	0.70	0.56	3.69	1.77	0.33	-1.10	1.77
$LaMnO_3$	$LaMO_3$	1.39	0.65	3.86	1.24	0.37	-0.49	1.24
$LaRuO_3$	$LaMO_3$	1.87	0.74	3.74	0.64	0.27	-0.10	0.64
CoO	MO	1.99	0.78	3.93	0.71	0.34	-0.02	0.71
Mn-FGM	SACs	1.89	0.88	4.08	0.96	0.37	-0.22	0.96
Fe-FGM	SACs	2.12	1.04	4.11	0.76	0.31	-0.15	0.76
PtO_2	Rutiles	2.35	1.21	4.22	0.64	0.28	-0.09	0.64

b) Materials with $n = 2$

Material	Family	ΔG_O	ΔG_{OH}	ΔG_{OOH}	η_{OER}	$\gamma_{OOH/OH}$	$\gamma_{O/OH}$	$\gamma_{OOH/O}$
Fe-TiO_2 (5cM)	u/d TiO_2	3.73	2.11	4.95	0.88	0.19	0.39	-0.01
Fe-TiO_2 (5cM)	u/d TiO_2	4.32	2.17	5.10	0.94	0.24	0.92	-0.44
Ir-TiO_2 (5cM)	u/d TiO_2	3.46	1.76	4.68	0.53	0.23	0.47	-0.00(47)
Ni-TiO_2 (5cM)	u/d TiO2	4.59	2.39	5.12	1.16	0.14	0.97	-0.69
Ni-BH_2	Porphyrins	3.85	1.93	4.79	0.70	0.20	0.69	-0.29
Cu-H	Porphyrins	4.12	2.07	5.07	0.84	0.27	0.82	-0.28
$SrNiO_3$	$SrMO_3$	3.85	2.18	4.93	0.95	0.15	0.44	-0.15
CuO	MO	3.93	2.00	4.76	0.77	0.15	0.70	-0.41
Ni-FGM	SACs	4.17	2.15	4.92	0.92	0.16	0.79	-0.48
Cu-FGM	SACs	4.52	2.32	5.12	1.09	0.17	0.98	-0.64
Pd-FGM	SACs	4.66	2.51	5.08	1.28	0.06	0.92	-0.80
Ag-FGM	SACs	4.77	2.53	5.13	1.30	0.07	1.01	-0.87
Pt-FGM	SACs	4.62	2.42	5.11	1.19	0.11	0.96	-0.74
Au-FGM	SACs	4.84	2.62	5.12	1.39	0.02	0.99	-0.96
TiO_2	u/d TiO_2	4.60	2.08	5.08	1.29	0.27	1.29	-0.74

Cr-TiO_2 (6cM)	u/d TiO_2	4.15	1.73	5.23	1.19	0.52	1.19	-0.14
Cr-TiO_2 (6cM)	u/d TiO_2	3.85	1.57	5.04	1.05	0.51	1.05	-0.03
Mn-TiO_2 (5cM)	u/d TiO_2	4.22	2.07	5.07	0.92	0.27	0.92	-0.37
Mn-TiO_2 (6cM)	u/d TiO_2	4.20	1.95	5.09	1.02	0.34	1.02	-0.33
Mn-TiO_2 (6cM)	u/d TiO_2	4.50	2.02	5.07	1.25	0.30	1.25	-0.65
Mn-TiO_2 (6cM)	u/d TiO_2	4.49	2.02	5.10	1.24	0.31	1.24	-0.61
Fe-TiO_2 (6cM)	u/d TiO_2	3.81	1.56	4.98	1.02	0.48	1.02	-0.05
Fe-TiO_2 (6cM)	u/d TiO_2	3.98	1.56	5.06	1.19	0.52	1.19	-0.14
Fe-TiO_2 (5cM)	u/d TiO_2	3.89	1.51	4.94	1.15	0.49	1.15	-0.17
Ru-TiO_2 (6cM)	u/d TiO_2	3.84	1.43	4.96	1.18	0.54	1.18	-0.10
Ru-TiO_2 (6cM)	u/d TiO_2	3.90	1.48	4.95	1.19	0.51	1.19	-0.17
Ir-TiO_2 (6cM)	u/d TiO_2	3.78	1.45	4.94	1.10	0.52	1.10	-0.06
Ir-TiO_2 (6cM)	u/d TiO_2	3.65	1.36	4.86	1.06	0.52	1.06	-0.01
Ni-TiO_2 (5cM)	u/d TiO_2	4.55	2.04	4.79	1.28	0.15	1.28	-0.98
Ni-TiO_2 (6cM)	u/d TiO_2	4.27	2.04	5.31	1.00	0.41	1.00	-0.18
Ni-TiO_2 (6cM)	u/d TiO_2	4.57	2.09	5.08	1.25	0.27	1.25	-0.71
Ni-TiO_2 (6cM)	u/d TiO_2	4.47	2.05	5.06	1.19	0.28	1.19	-0.63
Ni-H	Porphyrins	3.66	1.82	4.85	0.61	0.28	0.61	-0.04
Ni-CH_3	Porphyrins	3.82	1.84	4.60	0.75	0.15	0.75	-0.45
Cu-F	Porphyrins	4.15	2.04	5.02	0.88	0.26	0.88	-0.37
Cu-CH_3	Porphyrins	3.76	1.84	4.89	0.69	0.30	0.69	-0.10
Cu-BH_2	Porphyrins	4.61	1.78	4.99	1.61	0.37	1.61	-0.86
$SrScO_3$	$SrMO_3$	5.23	2.39	5.21	1.61	0.18	1.61	-1.26
$SrTiO_3$	$SrMO_3$	3.91	1.61	4.91	1.07	0.42	1.07	-0.23
$SrCuO_3$	$SrMO_3$	4.75	2.33	5.31	1.20	0.26	1.20	-0.67
$SrZnO_3$	$SrMO_3$	5.16	2.56	5.41	1.37	0.19	1.37	-0.99
$SrGeO_3$	$SrMO_3$	4.44	1.74	5.22	1.47	0.51	1.47	-0.45
$LaScO_3$	$LaMO_3$	4.80	1.83	4.88	1.75	0.30	1.75	-1.15
$LaCuO_3$	$LaMO_3$	4.92	2.42	5.39	1.27	0.26	1.27	-0.76
$LaZnO_3$	$LaMO_3$	5.18	2.48	5.28	1.47	0.17	1.47	-1.13
$LaGaO_3$	$LaMO_3$	4.89	1.96	5.02	1.70	0.30	1.70	-1.10
CaO	MO	5.38	2.33	5.04	1.83	0.13	1.83	-1.57
$BaNiO_3$	$BaNiO_x$	3.88	1.87	4.99	0.78	0.33	0.78	-0.12
$BaNiO_{3-d2}$	$BaNiO_x$	3.94	1.78	5.15	0.93	0.46	0.93	-0.02
LSNMR@Ni	LSNMR	3.71	1.47	4.41	1.01	0.24	1.01	-0.53
V-TiO_2 (5cM)	u/d TiO_2	2.35	1.38	4.49	0.92	0.33	-0.26	0.92
Cr-TiO_2 (5cM)	u/d TiO_2	2.43	1.86	4.87	1.22	0.28	-0.66	1.22
$LaCoO_3$	$LaMO_3$	2.22	1.49	4.51	1.05	0.28	-0.50	1.05
Ir-FGM	SACs	2.42	1.25	4.27	0.62	0.28	-0.06	0.62
V-TiO_2 (5cM)	u/d TiO_2	3.17	1.19	4.67	0.75	0.51	0.75	0.28
Nb-TiO_2 (5cM)	u/d TiO_2	2.14	0.24	3.74	0.67	0.52	0.67	0.38
Mo-TiO_2 (6cM)	u/d TiO_2	2.35	0.59	4.10	0.53	0.53	0.53	0.53
Ru-TiO_2 (6cM)	u/d TiO_2	2.98	0.50	4.53	1.25	0.79	1.25	0.33
$Sr_5Na_2Ru_8O_{24}$	$Sr_xNa_yRuO_3$	2.66	0.94	4.34	0.49	0.47	0.49	0.45
LSNMR@Ru	LSNMR	2.27	0.54	3.89	0.50	0.45	0.50	0.39
Mo-TiO_2 (6cM)	u/d TiO_2	2.19	0.63	4.17	0.76	0.54	0.33	0.76
W-TiO_2 (6cM)	u/d TiO_2	1.47	0.22	3.71	1.02	0.52	0.02	1.02
Ir-TiO_2 (6cM)	u/d TiO_2	2.73	1.14	4.64	0.69	0.52	0.36	0.69
Cu-NH_2	Porphyrins	2.70	0.92	4.49	0.56	0.55	0.55	0.56
FeO	MO	1.77	0.38	3.75	0.76	0.45	0.15	0.76
NiO	MO	2.49	1.04	4.12	0.40	0.31	0.22	0.40
Sr_2NiIrO_6	Sr2MIrO_6	2.12	0.77	3.25	0.44	0.01	0.12	-0.10
Nb-TiO_2 (6cM)	u/d TiO_2	2.39	0.06	3.59	1.10	0.54	1.10	-0.02
Ta-TiO_2 (6cM)	u/d TiO_2	2.17	-0.35	3.37	1.29	0.63	1.29	-0.02
Ta-TiO_2 (6cM)	u/d TiO_2	2.55	0.13	3.64	1.19	0.53	1.19	-0.13
Sr_2ScIrO_6	Sr_2MIrO_6	1.48	0.41	3.50	0.79	0.32	-0.16	0.79
Sr_2FeIrO_6	Sr_2MIrO_6	1.51	0.30	3.50	0.76	0.37	-0.02	0.76
W-TiO_2 (5cM)	u/d TiO_2	-0.33	-0.23	3.16	2.27	0.47	-1.33	2.27
W-TiO_2 (6cM)	u/d TiO_2	0.26	0.06	3.50	2.02	0.49	-1.03	2.02
W-TiO_2 (6cM)	u/d TiO_2	1.16	0.06	3.60	1.22	0.54	-0.13	1.22

Ir-TiO_2 (5cM)	u/d TiO_2	1.81	0.62	3.68	0.65	0.30	-0.04	0.65
Cr-H	Porphyrins	0.84	0.26	3.53	1.46	0.40	-0.65	1.46
Cr-F	Porphyrins	1.00	0.38	3.63	1.40	0.39	-0.61	1.40
Cr-OH	Porphyrins	0.76	0.22	3.49	1.50	0.41	-0.69	1.50
Cr-CH_3	Porphyrins	0.62	0.18	3.34	1.49	0.35	-0.79	1.49
Cr-BH_2	Porphyrins	0.94	0.36	3.58	1.41	0.38	-0.65	1.41
Cr-NH_2	Porphyrins	0.61	0.10	3.33	1.49	0.38	-0.73	1.49
Mn-H	Porphyrins	0.93	0.27	3.53	1.36	0.40	-0.57	1.36
Mn-OH	Porphyrins	0.74	0.22	3.51	1.54	0.42	-0.71	1.54
Mn-CH_3	Porphyrins	0.74	0.18	3.39	1.42	0.37	-0.67	1.42
Mn-NH_2	Porphyrins	0.46	-0.05	3.18	1.49	0.38	-0.72	1.49
Fe-OH	Porphyrins	1.08	0.48	3.62	1.32	0.34	-0.63	1.32
Fe-NH_2	Porphyrins	0.90	0.34	3.45	1.32	0.32	-0.67	1.32
$SrVO_3$	$SrMO_3$	-0.07	0.13	3.35	2.19	0.38	-1.43	2.19
$LaTiO_3$	$LaMO_3$	-1.82	-1.16	2.23	2.82	0.47	-1.89	2.82
$LaVO_3$	$LaMO_3$	-0.75	-0.22	2.96	2.48	0.36	-1.75	2.48
TiO	MO	-1.62	-1.06	2.32	2.71	0.46	-1.79	2.71
VO	MO	-0.95	-0.71	2.56	2.28	0.41	-1.46	2.28
CrO	MO	0.23	-0.14	2.90	1.45	0.29	-0.87	1.45
MnO	MO	1.43	0.42	3.67	1.01	0.39	-0.22	1.01
Cr-FGM	SACs	0.89	0.35	3.61	1.49	0.40	-0.69	1.49
Ru-FGM	SACs	1.72	0.58	3.64	0.68	0.30	-0.09	0.68
ScO	MO	-0.90	-1.52	1.98	1.71	0.52	-0.61	1.65

c) Materials with $n = 3$

Material	Family	ΔG_O	ΔG_{OH}	ΔG_{OOH}	η_{OER}	$\gamma_{OOH/OH}$	$\gamma_{O/OH}$	$\gamma_{OOH/O}$
Mn-TiO_2 (5cM)	u/d TiO_2	3.39	1.93	4.82	0.70	0.22	0.23	0.21
Ni-F	Porphyrins	3.43	1.86	4.94	0.63	0.31	0.35	0.27
$SrCoO_3$	$SrMO_3$	3.00	1.54	4.45	0.31	0.22	0.23	0.22
$BaNiO_{3-d1}$	$BaNiO_x$	3.54	1.93	5.00	0.70	0.31	0.38	0.23
$BaNiO_2$	$BaNiO_x$	3.46	1.85	4.78	0.62	0.24	0.38	0.09
Cr-TiO_2 (5cM)	u/d TiO_2	3.45	1.62	5.14	0.60	0.53	0.60	0.47
Ru-TiO_2 (5cM)	u/d TiO_2	3.55	1.74	5.12	0.58	0.46	0.58	0.35
Ni-OH	Porphyrins	3.22	1.58	4.79	0.40	0.37	0.40	0.34
Ni-NH_2	Porphyrins	3.10	1.32	4.46	0.55	0.34	0.55	0.13
Cu-OH	Porphyrins	3.69	1.62	4.94	0.84	0.43	0.84	0.02
$LaNiO_3$	$LaMO_3$	3.10	1.54	4.62	0.33	0.31	0.33	0.29
Rh-FGM	SACs	2.84	1.24	4.20	0.37	0.25	0.37	0.12
$Sr_{7/8}Na_{1/8}RuO_3$	$Sr_xNa_yRuO_3$	3.16	1.56	4.43	0.37	0.21	0.37	0.04
$Sr_6/_8Na_{2/8}RuO_3$	$Sr_xNa_yRuO_3$	3.31	1.59	4.76	0.49	0.36	0.49	0.22
$Sr_7Ru_8O_{24}$	$Sr_xNa_yRuO_3$	3.38	1.58	5.02	0.57	0.49	0.57	0.41
$Sr_6Na_1Ru_8O_{24}$	$Sr_xNa_yRuO_3$	3.44	1.62	4.79	0.59	0.36	0.59	0.12
Cr-TiO_2 (6cM)	u/d TiO_2	2.91	1.24	4.76	0.63	0.53	0.44	0.63
Co-BH_2	Porphyrins	2.48	1.24	4.29	0.58	0.30	0.01	0.58
$SrFeO_3$	$SrMO_3$	2.99	1.68	4.69	0.48	0.28	0.08	0.48
$LaFeO_3$	$LaMO_3$	2.75	1.25	4.55	0.57	0.42	0.27	0.57
RuO_2	Rutiles	2.72	1.35	4.35	0.40	0.27	0.14	0.40
MnO_2	Rutiles	3.20	1.84	5.04	0.61	0.37	0.13	0.61
Sr_2CoIrO_6	Sr_2MIrO_6	1.63	0.00	3.40	0.54	0.47	0.40	0.54
Nb-TiO_2 (6cM)	u/d TiO_2	2.34	0.11	3.61	1.00	0.52	1.00	0.05
Ta-TiO_2 (5cM)	u/d TiO_2	1.50	-0.51	3.04	0.78	0.55	0.78	0.32
Ta-TiO_2 (5cM)	u/d TiO_2	1.86	-0.02	3.52	0.65	0.54	0.65	0.44
Ta-TiO_2 (6cM)	u/d TiO_2	2.34	0.08	3.60	1.03	0.53	1.03	0.04
Nb-TiO_2 (5cM)	u/d TiO_2	1.39	-0.08	3.39	0.78	0.51	0.24	0.78
IrO_2	Rutiles	1.65	0.29	3.45	0.57	0.35	0.13	0.57

Table S2. δ optimized values of ΔG_O , ΔG_{OH}, ΔG_{OOH} (in eV), η_{OER} (in V), $\gamma_{OOH/OH}$, $\gamma_{OH/O}$ and $\gamma_{OOH/O}$ (in V) classified according to the number of electrochemical steps over 1.23 eV (n).

a) Materials with $n = 2$

Material	δ	$\Delta G_{O,\delta}$	$\Delta G_{OH,\delta}$	$\Delta G_{OOH,\delta}$	$\eta_{OER,\delta}$	$\gamma_{OOH/OH,\delta}$	$\gamma_{O/OH,\delta}$	$\gamma_{OOH/O,\delta}$
Fe-TiO_2 (5cM)	-0.44	2.85	1.67	4.51	0.44	0.19	-0.05	0.44
$SrNiO_3$	-0.55	2.75	1.63	4.38	0.40	0.15	-0.11	0.40
Pd-FGM	-1.04	2.57	1.47	4.04	0.24	0.06	-0.12	0.24
Ag-FGM	-1.09	2.60	1.44	4.04	0.21	0.07	-0.07	0.21
Pt-FGM	-0.96	2.69	1.46	4.15	0.23	0.11	-0.00(05)	0.23
Au-FGM	-1.18	2.49	1.45	3.94	0.22	0.02	-0.19	0.22
Cr-TiO_2 (5cM)	0.29	3.02	2.15	5.17	0.92	0.28	-0.36	0.92
$LaCoO_3$	0.40	3.02	1.89	4.90	0.66	0.28	-0.10	0.66
Mn-TiO_2 (5cM)	-0.25	2.90	1.68	4.58	0.45	0.22	-0.01	0.45
Sr_2MIrO_6	0.47	2.43	0.88	3.97	0.32	0.32	0.31	0.32
Sr_2MIrO_6	0.39	2.29	0.69	3.89	0.37	0.37	0.37	0.37
V-TiO_2 (6cM)	-0.60	2.19	0.42	3.96	0.54	0.54	0.54	0.54
V-TiO_2 (6cM)	-0.59	2.29	0.54	4.04	0.52	0.52	0.52	0.52
Co-FGM	-0.27	2.40	0.95	3.85	0.22	0.22	0.22	0.22
$LaRuO_3$	0.37	2.61	1.11	4.11	0.27	0.27	0.27	0.27
CoO	0.37	2.72	1.15	4.30	0.34	0.34	0.34	0.34
TiO_2	-1.02	2.56	1.06	4.06	0.27	0.27	0.27	0.27
Cr-TiO_2 (6cM)	-0.67	2.81	1.06	4.56	0.52	0.52	0.52	0.52
Cr-TiO_2 (6cM)	-0.54	2.76	1.02	4.50	0.51	0.51	0.51	0.51
Mn-TiO_2 (6cM)	-0.95	2.59	1.06	4.12	0.30	0.30	0.30	0.30
Mn-TiO_2 (6cM)	-0.93	2.63	1.09	4.17	0.31	0.31	0.31	0.31
Fe-TiO_2 (6cM)	-0.54	2.73	1.02	4.44	0.48	0.48	0.48	0.48
Fe-TiO_2 (6cM)	-0.67	2.64	0.89	4.39	0.52	0.52	0.52	0.52
Fe-TiO_2 (5cM)	-0.66	2.56	0.84	4.28	0.49	0.49	0.49	0.49
Ru-TiO_2 (6cM)	-0.64	2.55	0.78	4.32	0.54	0.54	0.54	0.54
Ru-TiO_2 (6cM)	-0.68	2.53	0.79	4.27	0.51	0.51	0.51	0.51
Ir-TiO_2 (6cM)	-0.58	2.61	0.86	4.36	0.52	0.52	0.52	0.52
Ir-TiO_2 (6cM)	-0.54	2.57	0.82	4.32	0.52	0.52	0.52	0.52
Ni-TiO_2 (6cM)	-0.98	2.60	1.10	4.10	0.27	0.27	0.27	0.27
Ni-TiO_2 (6cM)	-0.91	2.64	1.13	4.15	0.28	0.28	0.28	0.28
Cu-BH_2	-1.23	2.15	0.54	3.75	0.37	0.37	0.37	0.37
$SrScO_3$	-1.43	2.36	0.95	3.77	0.18	0.18	0.18	0.18
$SrTiO_3$	-0.65	2.60	0.96	4.25	0.42	0.42	0.42	0.42
$SrGeO_3$	-0.96	2.52	0.78	4.26	0.51	0.51	0.51	0.51
$LaZnO_3$	-1.30	2.58	1.18	3.98	0.17	0.17	0.17	0.17
V-TiO_2 (5cM)	-0.24	2.69	0.95	4.43	0.51	0.51	0.51	0.51
Mo-TiO_2 (6cM)	-0.00(39)	2.34	0.58	4.10	0.53	0.53	0.53	0.53
Ru-TiO_2 (6cM)	-0.46	2.05	0.03	4.07	0.79	0.79	0.79	0.79
$Sr_5Na_2Ru_8O_{24}$	-0.02	2.62	0.92	4.32	0.47	0.47	0.47	0.47
LSNMR@Ru	-0.06	2.16	0.48	3.83	0.45	0.45	0.44	0.45
Mo-TiO_2 (6cM)	0.21	2.61	0.84	4.38	0.54	0.54	0.54	0.54
W-TiO_2 (6cM)	0.50	2.46	0.71	4.21	0.52	0.52	0.52	0.52
Cu-NH_2	0.00(28)	2.71	0.92	4.49	0.55	0.55	0.55	0.55
FeO	0.30	2.37	0.68	4.05	0.45	0.45	0.45	0.45
NiO	0.09	2.67	1.13	4.21	0.31	0.31	0.31	0.31
W-TiO_2 (6cM)	0.67	2.50	0.73	4.27	0.54	0.54	0.54	0.54
Ir-TiO_2 (5cM)	0.34	2.49	0.96	4.02	0.30	0.30	0.30	0.30
Cr-NH_2	1.11	2.82	1.21	4.44	0.38	0.38	0.38	0.38
Mn-CH_3	1.04	2.83	1.23	4.44	0.37	0.37	0.37	0.37
Mn-NH_2	1.11	2.67	1.06	4.29	0.38	0.38	0.38	0.38
$LaTiO_3$	2.36	2.90	1.20	4.59	0.47	0.47	0.47	0.47
TiO	2.25	2.88	1.19	4.57	0.46	0.46	0.46	0.46
VO	1.87	2.79	1.16	4.43	0.41	0.41	0.41	0.41

CrO	1.16	2.54	1.02	4.06	0.29	0.29	0.29	0.29
MnO	0.62	2.66	1.04	4.29	0.39	0.39	0.39	0.39
Ru-FGM	0.39	2.50	0.97	4.02	0.30	0.30	0.30	0.30
Ni-NH_2	-0.21	2.68	1.11	4.25	0.34	0.34	0.34	0.34
Cu-OH	-0.41	2.87	1.21	4.53	0.43	0.43	0.43	0.43
Rh-FGM	-0.13	2.59	1.11	4.07	0.25	0.25	0.25	0.25
Sr_2MIrO_6	0.16	2.44	0.93	3.41	0.28	0.01	0.28	-0.26

b) Materials with $n = 3$

Material	δ	$\Delta G_{O,\delta}$	$\Delta G_{OH,\delta}$	$\Delta G_{OOH,\delta}$	$\eta_{OER,\delta}$	$\gamma_{OOH/OH,\delta}$	$\gamma_{O/OH,\delta}$	$\gamma_{OOH/O,\delta}$
Mo-TiO_2 (5cM)	1.40	3.30	1.83	5.13	0.60	0.42	0.24	0.60
Mo-TiO_2 (5cM)	0.92	3.31	1.54	5.08	0.54	0.54	0.54	0.54
Mo-TiO_2 (6cM)	1.20	3.26	1.49	5.03	0.54	0.54	0.54	0.54
W-TiO_2 (5cM)	1.57	3.56	1.92	5.47	0.69	0.55	0.41	0.69
Ru-TiO_2 (5cM)	0.48	3.10	1.53	4.67	0.34	0.34	0.34	0.34
Mn-F	0.95	3.04	1.42	4.66	0.39	0.39	0.39	0.39
Mn-BH_2	0.89	3.19	1.64	4.83	0.41	0.37	0.33	0.41
Fe-H	0.98	3.15	1.61	4.75	0.38	0.34	0.31	0.38
Fe-F	0.90	3.17	1.58	4.75	0.36	0.36	0.36	0.36
Fe-CH_3	1.02	3.15	1.60	4.75	0.37	0.35	0.33	0.37
Fe-BH_2	0.85	3.21	1.73	4.94	0.50	0.38	0.26	0.50
Co-H	0.29	2.88	1.50	4.38	0.27	0.21	0.15	0.27
Co-F	0.54	3.05	1.58	4.63	0.35	0.29	0.24	0.35
Co-OH	0.71	3.07	1.59	4.67	0.36	0.31	0.25	0.36
Co-CH_3	0.39	2.93	1.40	4.47	0.30	0.30	0.30	0.30
Co-NH_2	0.91	3.09	1.68	4.77	0.45	0.31	0.17	0.45
$SrCrO_3$	1.16	3.24	1.91	5.15	0.68	0.39	0.10	0.68
$SrMnO_3$	0.45	3.20	1.66	4.86	0.43	0.37	0.31	0.43
$SrRuO_3$	0.33	2.93	1.45	4.42	0.26	0.26	0.26	0.26
$LaCrO_3$	1.22	3.13	1.78	4.91	0.55	0.33	0.12	0.55
$LaMnO_3$	0.87	3.12	1.52	4.73	0.37	0.37	0.37	0.37
Mn-FGM	0.59	3.07	1.48	4.67	0.37	0.37	0.37	0.37
Fe-FGM	0.45	3.02	1.49	4.56	0.31	0.31	0.31	0.31
PtO_2	0.33	3.01	1.54	4.55	0.31	0.28	0.24	0.31
Fe-TiO_2 (5cM)	-0.69	2.94	1.48	4.41	0.25	0.24	0.23	0.25
Ir-TiO_2 (5cM)	-0.27	2.93	1.49	4.42	0.26	0.23	0.21	0.26
Ni-TiO_2 (5cM)	-0.93	2.74	1.46	4.20	0.23	0.14	0.05	0.23
Ni-BH_2	-0.50	2.86	1.43	4.29	0.20	0.20	0.20	0.20
Cu-H	-0.56	3.00	1.51	4.51	0.28	0.27	0.26	0.28
CuO	-0.59	2.75	1.41	4.17	0.18	0.15	0.11	0.18
Ni-FGM	-0.70	2.77	1.45	4.22	0.22	0.16	0.09	0.22
Cu-FGM	-0.86	2.80	1.45	4.25	0.22	0.17	0.12	0.22
Mn-TiO_2 (5cM)	-0.65	2.92	1.42	4.42	0.27	0.27	0.27	0.27
Mn-TiO_2 (6cM)	-0.68	2.84	1.27	4.41	0.34	0.34	0.34	0.34
Ni-TiO_2 (6cM)	-0.59	3.08	1.44	4.72	0.41	0.41	0.41	0.41
Ni-H	-0.32	3.01	1.50	4.53	0.28	0.28	0.28	0.28
Ni-CH_3	-0.60	2.62	1.24	4.00	0.15	0.15	0.15	0.15
Cu-F	-0.62	2.91	1.42	4.39	0.26	0.26	0.26	0.26
Cu-CH_3	-0.39	2.97	1.44	4.50	0.30	0.30	0.30	0.30
$SrCuO_3$	-0.93	2.88	1.39	4.38	0.26	0.26	0.26	0.26
$SrZnO_3$	-1.18	2.80	1.38	4.23	0.19	0.19	0.19	0.19
$LaCuO_3$	-1.02	2.89	1.41	4.38	0.26	0.26	0.26	0.26
$BaNiO_3$	-0.45	2.98	1.42	4.54	0.33	0.33	0.33	0.33
$BaNiO_{3\text{-d2}}$	-0.48	2.99	1.30	4.67	0.46	0.46	0.45	0.46
V-TiO_2 (5cM)	0.38	3.12	1.76	4.88	0.53	0.33	0.13	0.53
Ir-FGM	0.30	3.02	1.55	4.57	0.32	0.28	0.24	0.32
Ir-TiO_2 (6cM)	0.16	3.05	1.30	4.80	0.52	0.52	0.52	0.52
W-TiO_2 (5cM)	1.80	3.26	1.56	4.96	0.47	0.47	0.47	0.47

W-TiO_2 (6cM)	1.52	3.30	1.58	5.02	0.49	0.49	0.49	0.49
Cr-H	1.05	2.95	1.32	4.58	0.40	0.40	0.40	0.40
Cr-F	1.00	3.01	1.38	4.63	0.39	0.39	0.39	0.39
Cr-OH	1.09	2.95	1.32	4.59	0.41	0.41	0.41	0.41
Cr-CH_3	1.14	2.90	1.32	4.48	0.35	0.35	0.35	0.35
Cr-BH_2	1.03	3.00	1.39	4.61	0.38	0.38	0.38	0.38
Mn-H	0.97	2.87	1.24	4.49	0.40	0.40	0.40	0.40
Mn-OH	1.13	2.99	1.35	4.64	0.42	0.42	0.42	0.42
Fe-OH	0.97	3.02	1.45	4.60	0.34	0.34	0.34	0.34
Fe-NH_2	1.00	2.89	1.34	4.44	0.32	0.32	0.32	0.32
$SrVO_3$	1.65	3.22	1.78	5.00	0.55	0.38	0.21	0.55
$LaVO_3$	1.97	3.18	1.74	4.93	0.51	0.36	0.21	0.51
Cr-FGM	1.09	3.07	1.44	4.69	0.40	0.40	0.40	0.40
Ni-F	-0.18	3.08	1.68	4.76	0.45	0.31	0.17	0.45
$SrCoO_3$	-0.05	2.91	1.49	4.40	0.26	0.22	0.19	0.26
$BaNiO_{3-d1}$	-0.24	3.07	1.69	4.76	0.47	0.31	0.14	0.47
$BaNiO_2$	-0.27	2.93	1.58	4.51	0.36	0.24	0.11	0.36
Cr-TiO_2 (5cM)	-0.07	3.31	1.55	5.07	0.53	0.53	0.53	0.53
Ru-TiO_2 (5cM)	-0.12	3.31	1.62	5.00	0.46	0.46	0.46	0.46
Ni-OH	-0.03	3.15	1.55	4.76	0.37	0.37	0.37	0.37
$LaNiO_3$	-0.02	3.06	1.52	4.60	0.31	0.31	0.31	0.31
$Sr_{7/8}$ $Na_{1/8}RuO_3$	-0.17	2.83	1.39	4.26	0.21	0.21	0.20	0.21
$Sr_{6/8}Na_{2/8}RuO_3$	-0.14	3.04	1.45	4.62	0.36	0.36	0.35	0.36
$Sr_7Ru_8O_{24}$	-0.08	3.22	1.50	4.94	0.49	0.49	0.49	0.49
$Sr_6Na_1Ru_8O_{24}$	-0.24	2.97	1.38	4.55	0.36	0.36	0.35	0.36
Cr-TiO_2 (6cM)	0.09	3.09	1.33	4.85	0.53	0.53	0.53	0.53
Co-BH_2	0.29	3.05	1.53	4.58	0.30	0.30	0.30	0.30
$SrFeO_3$	0.02	3.02	1.69	4.71	0.46	0.28	0.10	0.46
$LaFeO_3$	0.15	3.05	1.40	4.70	0.42	0.42	0.42	0.42
RuO_2	0.13	2.98	1.48	4.48	0.27	0.27	0.27	0.27
MnO_2	0.00	3.20	1.84	5.04	0.61	0.37	0.13	0.61
V-TiO_2 (6cM)	-0.58	1.60	-0.19	3.39	0.56	0.56	0.56	0.56
Nb-TiO_2 (6cM)	-0.64	1.41	-0.35	3.16	0.53	0.52	0.53	0.52
LSNMR@Mn	-0.60	1.92	0.36	3.48	0.33	0.33	0.33	0.33
Ni-TiO_2 (5cM)	-1.13	2.28	0.90	3.66	0.15	0.15	0.15	0.15
$LaScO_3$	-1.45	1.91	0.38	3.43	0.30	0.30	0.30	0.30
$LaGaO_3$	-1.40	2.09	0.56	3.63	0.30	0.30	0.30	0.30
CaO	-1.59	2.20	0.73	3.45	0.24	0.13	0.24	0.02
LSNMR@Ni	-0.77	2.17	0.70	3.64	0.24	0.24	0.24	0.24
Nb-TiO_2 (5cM)	-0.15	1.84	0.09	3.59	0.52	0.52	0.52	0.52
Nb-TiO_2 (6cM)	-0.50	1.38	-0.45	3.09	0.60	0.54	0.60	0.48
Ta-TiO_2 (6cM)	-0.49	1.19	-0.84	2.89	0.80	0.63	0.80	0.46
Ta-TiO_2 (6cM)	-0.57	1.40	-0.45	3.07	0.62	0.53	0.62	0.44
ScO	1.16	1.42	-0.35	3.14	0.55	0.52	0.55	0.49
Sr_2MIrO_6	0.07	1.77	0.07	3.47	0.47	0.47	0.47	0.47
Nb-TiO_2 (6cM)	-0.46	1.41	-0.36	3.15	0.54	0.52	0.54	0.51
Ta-TiO_2 (5cM)	-0.07	1.36	-0.58	2.98	0.71	0.55	0.71	0.38
Ta-TiO_2 (5cM)	-0.11	1.64	-0.13	3.41	0.54	0.54	0.54	0.54
Ta-TiO_2 (6cM)	-0.47	1.39	-0.40	3.13	0.56	0.53	0.56	0.51
Nb-TiO_2 (5cM)	0.27	1.92	0.18	3.66	0.51	0.51	0.51	0.51
IrO_2	0.22	2.09	0.51	3.67	0.35	0.35	0.35	0.35

Table S3. ε optimized values of ΔG_O , ΔG_{OH}, ΔG_{OOH} (in eV), η_{OER} (in V), $\gamma_{OOH/OH}$, $\gamma_{OH/O}$ and $\gamma_{OOH/O}$ (in V) classified according to the number of electrochemical steps over 1.23 eV (n).

a) Materials with $n = 1$

Material	ε	$\Delta G_{O,\varepsilon}$	$\Delta G_{OH,\varepsilon}$	$\Delta G_{OOH,\varepsilon}$	$\eta_{OER,\varepsilon}$	$\gamma_{OOH/OH,\varepsilon}$	$\gamma_{O/OH,\varepsilon}$	$\gamma_{OOH/O,\varepsilon}$
V-TiO_2 (6cM)	0.00	2.76	0.39	3.97	1.14	0.56	1.14	-0.01
V-TiO_2 (6cM)	0.00	3.40	1.03	4.56	1.14	0.54	1.14	-0.06
V-TiO_2 (6cM)	0.00	3.47	1.13	4.63	1.11	0.52	1.11	-0.06
Nb-TiO_2 (6cM)	0.00	2.70	0.30	3.80	1.17	0.52	1.17	-0.12
Co-FGM	0.00	2.94	1.22	4.11	0.49	0.22	0.49	-0.05
LSNMR@Mn	0.00	3.12	0.96	4.08	0.93	0.33	0.93	-0.27

b) Materials with $n = 2$

Material	ε	$\Delta G_{O,\varepsilon}$	$\Delta G_{OH,\varepsilon}$	$\Delta G_{OOH,\varepsilon}$	$\eta_{OER,\varepsilon}$	$\gamma_{OOH/OH,\varepsilon}$	$\gamma_{O/OH,\varepsilon}$	$\gamma_{OOH/O,\varepsilon}$
TiO_2	0.00	4.60	2.08	5.08	1.29	0.27	1.29	-0.74
Cr-TiO_2 (6cM)	0.00	4.15	1.73	5.23	1.19	0.52	1.19	-0.14
Cr-TiO_2 (6cM)	0.00	3.85	1.57	5.04	1.05	0.51	1.05	-0.03
Mn-TiO_2 (5cM)	0.00	4.22	2.07	5.07	0.92	0.27	0.92	-0.37
Mn-TiO_2 (6cM)	0.00	4.20	1.95	5.09	1.02	0.34	1.02	-0.33
Mn-TiO_2 (6cM)	0.00	4.50	2.02	5.07	1.25	0.30	1.25	-0.65
Mn-TiO_2 (6cM)	0.00	4.49	2.02	5.10	1.24	0.31	1.24	-0.61
Fe-TiO_2 (5cM)	0.00	3.73	2.11	4.95	0.88	0.19	0.39	-0.00(47)
Fe-TiO_2 (5cM)	0.00	4.32	2.17	5.10	0.94	0.24	0.92	-0.44
Fe-TiO_2 (6cM)	0.00	3.81	1.56	4.98	1.02	0.48	1.02	-0.05
Fe-TiO_2 (6cM)	0.00	3.98	1.56	5.06	1.19	0.52	1.19	-0.14
Fe-TiO_2 (5cM)	0.00	3.89	1.51	4.94	1.15	0.49	1.15	-0.17
Ru-TiO_2 (6cM)	0.00	3.84	1.43	4.96	1.18	0.54	1.18	-0.10
Ru-TiO_2 (6cM)	0.00	3.90	1.48	4.95	1.19	0.51	1.19	-0.17
Ir-TiO_2 (5cM)	0.00	3.46	1.76	4.68	0.53	0.23	0.47	-0.00(47)
Ir-TiO_2 (6cM)	0.00	3.78	1.45	4.94	1.10	0.52	1.10	-0.06
Ir-TiO_2 (6cM)	0.00	3.65	1.36	4.86	1.06	0.52	1.06	-0.01
Ni-TiO_2 (5cM)	0.00	4.55	2.04	4.79	1.28	0.15	1.28	-0.98
Ni-TiO_2 (5cM)	0.00	4.59	2.39	5.12	1.16	0.14	0.97	-0.69
Ni-TiO_2 (6cM)	0.00	4.27	2.04	5.31	1.00	0.41	1.00	-0.18
Ni-TiO_2 (6cM)	0.00	4.57	2.09	5.08	1.25	0.27	1.25	-0.71
Ni-TiO_2 (6cM)	0.00	4.47	2.05	5.06	1.19	0.28	1.19	-0.63
Ni-H	0.00	3.66	1.82	4.85	0.61	0.28	0.61	-0.04
Ni-CH_3	0.00	3.82	1.84	4.60	0.75	0.15	0.75	-0.45
Ni-BH_2	0.00	3.85	1.93	4.79	0.70	0.20	0.69	-0.29
Cu-H	0.00	4.12	2.07	5.07	0.84	0.27	0.82	-0.28
Cu-F	0.00	4.15	2.04	5.02	0.88	0.26	0.88	-0.37
Cu-CH_3	0.00	3.76	1.84	4.89	0.69	0.30	0.69	-0.10
Cu-BH_2	0.00	4.61	1.78	4.99	1.61	0.37	1.61	-0.86
$SrScO_3$	0.00	5.23	2.39	5.21	1.61	0.18	1.61	-1.26
$SrTiO_3$	0.00	3.91	1.61	4.91	1.07	0.42	1.07	-0.23
$SrNiO_3$	0.00	3.85	2.18	4.93	0.95	0.15	0.44	-0.15
$SrCuO_3$	0.00	4.75	2.33	5.31	1.20	0.26	1.20	-0.67
$SrZnO_3$	0.00	5.16	2.56	5.41	1.37	0.19	1.37	-0.99
$SrGeO_3$	0.00	4.44	1.74	5.22	1.47	0.51	1.47	-0.45
$LaScO_3$	0.00	4.80	1.83	4.88	1.75	0.30	1.75	-1.15
$LaCuO_3$	0.00	4.92	2.42	5.39	1.27	0.26	1.27	-0.76
$LaZnO_3$	0.00	5.18	2.48	5.28	1.47	0.17	1.47	-1.13
$LaGaO_3$	0.00	4.89	1.96	5.02	1.70	0.30	1.70	-1.10
CaO	0.00	5.38	2.33	5.04	1.83	0.13	1.83	-1.57
CuO	0.00	3.93	2.00	4.76	0.77	0.15	0.70	-0.41

Ni-FGM	0.00	4.17	2.15	4.92	0.92	0.16	0.79	-0.48
Cu-FGM	0.00	4.52	2.32	5.12	1.09	0.17	0.98	-0.64
Pd-FGM	0.00	4.66	2.51	5.08	1.28	0.06	0.92	-0.80
Ag-FGM	0.00	4.77	2.53	5.13	1.30	0.07	1.01	-0.87
Pt-FGM	0.00	4.62	2.42	5.11	1.19	0.11	0.96	-0.74
Au-FGM	0.00	4.84	2.62	5.12	1.39	0.02	0.99	-0.96
$BaNiO_3$	0.00	3.88	1.87	4.99	0.78	0.33	0.78	-0.12
$BaNiO_{3\text{-}d2}$	0.00	3.94	1.78	5.15	0.93	0.46	0.93	-0.02
LSNMR@Ni	0.00	3.71	1.47	4.41	1.01	0.24	1.01	-0.53
V-TiO_2 (5cM)	-0.77	2.35	1.38	3.72	0.15	-0.06	-0.26	0.15
Cr-TiO_2 (5cM)	-0.59	2.43	1.86	4.28	0.63	-0.02	-0.66	0.63
$LaCoO_3$	-0.79	2.22	1.49	3.71	0.26	-0.12	-0.50	0.26
Cu-NH_2	-0.01	2.70	0.92	4.48	0.55	0.55	0.55	0.55
V-TiO_2 (5cM)	0.00	3.17	1.19	4.67	0.75	0.51	0.75	0.28
Nb-TiO_2 (5cM)	0.00	2.14	0.24	3.74	0.67	0.52	0.67	0.38
Mo-TiO_2 (6cM)	0.00	2.35	0.59	4.10	0.53	0.53	0.53	0.53
Ru-TiO_2 (6cM)	0.00	2.98	0.50	4.53	1.25	0.79	1.25	0.33
Ir-TiO_2 (6cM)	-0.32	2.73	1.14	4.32	0.36	0.36	0.36	0.36
NiO	-0.17	2.49	1.04	3.95	0.22	0.22	0.22	0.22
$Sr_5Na_2Ru_8O_{24}$	0.00	2.66	0.94	4.34	0.49	0.47	0.49	0.45
LSNMR@Ru	0.00	2.27	0.54	3.89	0.50	0.45	0.50	0.39
Mo-TiO_2 (6cM)	-0.42	2.19	0.63	3.75	0.33	0.33	0.33	0.33
Sr_2NiIrO_6	0.00	2.12	0.77	3.25	0.44	0.01	0.12	-0.10
Nb-TiO_2 (6cM)	0.00	2.39	0.06	3.59	1.10	0.54	1.10	-0.02
Ta-TiO_2 (6cM)	0.00	2.17	-0.35	3.37	1.29	0.63	1.29	-0.02
Ta-TiO_2 (6cM)	0.00	2.55	0.13	3.64	1.19	0.53	1.19	-0.13
Sr_2ScIrO_6	-0.30	1.48	0.41	3.20	0.49	0.17	-0.16	0.49
Sr_2FeIrO_6	-0.29	1.51	0.30	3.22	0.48	0.23	-0.02	0.48
Mo-TiO_2 (5cM)	-1.02	0.49	0.43	2.70	0.99	-0.09	-1.17	0.99
Mo-TiO_2 (5cM)	-0.97	1.47	0.62	3.19	0.50	0.06	-0.38	0.50
Mo-TiO_2 (6cM)	-0.94	0.86	0.29	2.89	0.80	0.07	-0.66	0.80
W-TiO_2 (5cM)	-1.23	0.42	0.35	2.67	1.02	-0.07	-1.16	1.02
Ru-TiO_2 (5cM)	-0.66	2.15	1.06	3.53	0.16	0.01	-0.14	0.16
Mn-F	-0.68	1.13	0.46	3.03	0.66	0.05	-0.56	0.66
Mn-BH_2	-0.78	1.42	0.75	3.17	0.52	-0.02	-0.56	0.52
Fe-H	-0.72	1.19	0.63	3.05	0.64	-0.02	-0.67	0.64
Fe-F	-0.71	1.37	0.68	3.14	0.55	0.00(32)	-0.54	0.55
Fe-CH_3	-0.72	1.11	0.57	3.01	0.68	-0.01	-0.70	0.68
Fe-BH_2	-0.87	1.51	0.88	3.22	0.47	-0.06	-0.59	0.47
Co-H	-0.48	2.30	1.21	3.61	0.08	-0.03	-0.14	0.08
Co-F	-0.64	1.97	1.04	3.44	0.25	-0.03	-0.30	0.25
Co-OH	-0.67	1.65	0.88	3.29	0.40	-0.03	-0.46	0.40
Co-CH_3	-0.54	2.15	1.01	3.53	0.16	0.03	-0.09	0.16
Co-NH_2	-0.77	1.26	0.77	3.09	0.60	-0.07	-0.74	0.60
$SrCrO_3$	-1.07	0.92	0.76	2.92	0.77	-0.15	-1.06	0.77
$SrMnO_3$	-0.80	2.30	1.21	3.61	0.08	-0.03	-0.14	0.08
$SrRuO_3$	-0.49	2.26	1.11	3.59	0.10	0.01	-0.08	0.10
$LaCrO_3$	-0.88	0.70	0.56	2.81	0.88	-0.11	-1.10	0.88
$LaMnO_3$	-0.70	1.39	0.65	3.16	0.53	0.02	-0.49	0.53
$LaRuO_3$	-0.35	1.87	0.74	3.40	0.29	0.10	-0.10	0.29
CoO	-0.48	1.99	0.78	3.45	0.24	0.11	-0.02	0.24
Mn-FGM	-0.67	1.89	0.88	3.41	0.28	0.03	-0.22	0.28
Fe-FGM	-0.59	2.12	1.04	3.52	0.17	0.01	-0.15	0.17
PtO_2	-0.59	2.35	1.21	3.64	0.06	-0.02	-0.09	0.05
W-TiO_2 (5cM)	-0.87	-0.33	-0.23	2.29	1.40	0.03	-1.33	1.40
W-TiO_2 (6cM)	-0.91	0.26	0.06	2.59	1.10	0.04	-1.03	1.10
W-TiO_2 (6cM)	-0.56	1.16	0.06	3.04	0.65	0.26	-0.13	0.65
Ir-TiO_2 (5cM)	-0.32	1.81	0.62	3.36	0.33	0.14	-0.04	0.33
Cr-H	-0.65	0.84	0.26	2.88	0.81	0.08	-0.65	0.81
Cr-F	-0.67	1.00	0.38	2.96	0.73	0.06	-0.61	0.73

Cr-OH	-0.65	0.76	0.22	2.84	0.85	0.08	-0.69	0.85
Cr-CH_3	-0.57	0.62	0.18	2.77	0.92	0.06	-0.79	0.92
Cr-BH_2	-0.65	0.94	0.36	2.93	0.76	0.06	-0.65	0.76
Cr-NH_2	-0.56	0.61	0.10	2.76	0.93	0.10	-0.73	0.93
Mn-H	-0.60	0.93	0.27	2.93	0.76	0.10	-0.57	0.76
Mn-OH	-0.68	0.74	0.22	2.83	0.86	0.07	-0.71	0.86
Mn-CH_3	-0.56	0.74	0.18	2.83	0.86	0.09	-0.67	0.86
Mn-NH_2	-0.49	0.46	-0.05	2.69	1.00	0.14	-0.72	1.00
Fe-OH	-0.62	1.08	0.48	3.00	0.69	0.03	-0.63	0.69
Fe-NH_2	-0.54	0.90	0.34	2.91	0.78	0.05	-0.67	0.78
$SrVO_3$	-0.93	-0.07	0.13	2.42	1.27	-0.08	-1.43	1.27
$LaTiO_3$	-0.68	-1.82	-1.16	1.55	2.14	0.12	-1.89	2.14
$LaVO_3$	-0.87	-0.75	-0.22	2.09	1.60	-0.07	-1.75	1.60
ScO	0.00	-0.90	-1.52	1.98	1.71	0.52	-0.61	1.65
TiO	-0.67	-1.62	-1.06	1.65	2.04	0.12	-1.79	2.04
VO	-0.57	-0.95	-0.71	1.99	1.70	0.12	-1.46	1.70
CrO	-0.33	0.23	-0.14	2.57	1.12	0.12	-0.87	1.12
MnO	-0.49	1.43	0.42	3.17	0.52	0.15	-0.22	0.52
Cr-FGM	-0.70	0.89	0.35	2.91	0.78	0.05	-0.69	0.78
Ru-FGM	-0.31	1.72	0.58	3.32	0.37	0.14	-0.09	0.37

c) Materials with $n = 3$

Material	ε	$\Delta G_{O,\varepsilon}$	$\Delta G_{OH,\varepsilon}$	$\Delta G_{OOH,\varepsilon}$	$\eta_{OER,\varepsilon}$	$\gamma_{OOH/OH,\varepsilon}$	$\gamma_{O/OH,\varepsilon}$	$\gamma_{OOH/O,\varepsilon}$
Co-BH_2	-0.57	2.48	1.24	3.72	0.01	0.01	0.01	0.01
$SrFeO_3$	-0.03	2.99	1.68	4.66	0.45	0.26	0.08	0.45
$LaFeO_3$	-0.30	2.75	1.25	4.25	0.27	0.27	0.27	0.27
RuO_2	-0.26	2.72	1.35	4.09	0.14	0.14	0.14	0.14
MnO_2	0.00	3.20	1.84	5.04	0.61	0.37	0.13	0.61
Cr-TiO_2 (5cM)	0.00	3.45	1.62	5.14	0.60	0.53	0.60	0.47
Cr-TiO_2 (6cM)	-0.18	2.91	1.24	4.58	0.44	0.44	0.44	0.44
Mn-TiO_2 (5cM)	0.00	3.39	1.93	4.82	0.70	0.22	0.23	0.21
Ru-TiO_2 (5cM)	0.00	3.55	1.74	5.12	0.58	0.46	0.58	0.35
Ni-F	0.00	3.43	1.86	4.94	0.63	0.31	0.35	0.27
Ni-OH	0.00	3.22	1.58	4.79	0.40	0.37	0.40	0.34
Ni-NH_2	0.00	3.10	1.32	4.46	0.55	0.34	0.55	0.13
Cu-OH	0.00	3.69	1.62	4.94	0.84	0.43	0.84	0.02
$SrCoO_3$	0.00	3.00	1.54	4.45	0.31	0.22	0.23	0.22
$LaNiO_3$	0.00	3.10	1.54	4.62	0.33	0.31	0.33	0.29
Rh-FGM	0.00	2.84	1.24	4.20	0.37	0.25	0.37	0.12
$BaNiO_{3\text{-}d1}$	0.00	3.54	1.93	5.00	0.70	0.31	0.38	0.23
$BaNiO_2$	0.00	3.46	1.85	4.78	0.62	0.24	0.38	0.09
$Sr_{7/8}Na_{1/8}RuO_3$	0.00	3.16	1.56	4.43	0.37	0.21	0.37	0.04
$Sr_{6/8}Na_{2/8}RuO_3$	0.00	3.31	1.59	4.76	0.49	0.36	0.49	0.22
$Sr_7Ru_8O_{24}$	0.00	3.38	1.58	5.02	0.57	0.49	0.57	0.41
$Sr_6Na_1Ru_8O_{24}$	0.00	3.44	1.62	4.79	0.59	0.36	0.59	0.12
Sr_2CoIrO_6	-0.13	1.63	0.00	3.28	0.42	0.41	0.40	0.42
W-TiO_2 (6cM)	-0.52	1.47	0.22	3.19	0.50	0.26	0.02	0.50
FeO	-0.41	1.77	0.38	3.34	0.35	0.25	0.15	0.35
Nb-TiO_2 (5cM)	-0.24	1.39	-0.08	3.15	0.54	0.39	0.24	0.54
Nb-TiO_2 (6cM)	0.00	2.34	0.11	3.61	1.00	0.52	1.00	0.05
Ta-TiO_2 (5cM)	0.00	1.50	-0.51	3.04	0.78	0.55	0.78	0.32
Ta-TiO_2 (5cM)	0.00	1.86	-0.02	3.52	0.65	0.54	0.65	0.44
Ta-TiO_2 (6cM)	0.00	2.34	0.08	3.60	1.03	0.53	1.03	0.04
IrO_2	-0.17	1.65	0.29	3.29	0.41	0.27	0.13	0.41
Ir-FGM	-0.60	2.42	1.25	3.67	0.02	-0.02	-0.06	0.02

Table S4. $\delta + \varepsilon$ optimized values of ΔG_O , ΔG_{OH}, ΔG_{OOH} (in eV), η_{OER} (in V), $\gamma_{OOH/OH}$, $\gamma_{OH/O}$ and $\gamma_{OOH/O}$ (in V) classified according to the number of electrochemical steps over 1.23 eV (n).

a) Materials with $n = 1$

Material	δ	ε	$\Delta G_{O,\delta+\varepsilon}$	$\Delta G_{OH,\delta+\varepsilon}$	$\Delta G_{OOH,\delta+e}$	$\eta_{OER,\delta+\varepsilon}$	$\gamma_{OOH/OH,\delta+\varepsilon}$	$\gamma_{O/OH,\delta+\varepsilon}$	$\gamma_{OOH/O,\delta+\varepsilon}$
LSNMR@Ni	-0.30	0.00	3.11	1.17	4.11	0.71	0.24	0.71	-0.23

b) Materials with $n = 2$

Material	δ	ε	$\Delta G_{O,\delta+}$	$\Delta G_{OH,\delta+\varepsilon}$	$\Delta G_{OOH,\delta+e}$	$\eta_{OER,\delta+\varepsilon}$	$\gamma_{OOH/OH,\delta+\varepsilon}$	$\gamma_{O/OH,\delta+\varepsilon}$	$\gamma_{OOH/O,\delta+}$
Pd-FGM	-0.30	0.00	4.06	2.21	4.78	0.98	0.06	0.62	-0.50
Ag-FGM	-0.30	0.00	4.17	2.23	4.83	1.00	0.07	0.71	-0.57
Pt-FGM	-0.30	0.00	4.02	2.12	4.81	0.89	0.11	0.66	-0.44
Au-FGM	-0.30	0.00	4.24	2.32	4.82	1.09	0.02	0.69	-0.66
TiO_2	-0.30	0.00	4.00	1.78	4.78	0.99	0.27	0.99	-0.44
Mn-TiO_2 (6cM)	-0.30	0.00	3.90	1.72	4.77	0.95	0.30	0.95	-0.35
Mn-TiO_2 (6cM)	-0.30	0.00	3.89	1.72	4.80	0.94	0.31	0.94	-0.31
Ni-TiO_2 (6cM)	-0.30	0.00	3.97	1.79	4.78	0.95	0.27	0.95	-0.41
Ni-TiO_2 (6cM)	-0.30	0.00	3.87	1.75	4.76	0.89	0.28	0.89	-0.33
Cu-BH_2	-0.30	0.00	4.01	1.48	4.69	1.31	0.37	1.31	-0.56
$SrScO_3$	-0.30	0.00	4.63	2.09	4.91	1.31	0.18	1.31	-0.96
$SrGeO_3$	-0.30	0.00	3.84	1.44	4.92	1.17	0.51	1.17	-0.15
$LaZnO_3$	-0.30	0.00	4.58	2.18	4.98	1.17	0.17	1.17	-0.83
Fe-TiO_2 (5cM)	-0.30	0.00	3.72	1.87	4.80	0.64	0.24	0.62	-0.14
Ni-TiO_2 (5cM)	-0.30	0.00	3.99	2.09	4.82	0.86	0.14	0.67	-0.39
CuO	-0.30	0.00	3.33	1.70	4.46	0.47	0.15	0.40	-0.11
Ni-FGM	-0.30	0.00	3.57	1.85	4.62	0.62	0.16	0.49	-0.18
Cu-FGM	-0.30	0.00	3.92	2.02	4.82	0.79	0.17	0.68	-0.34
Mn-TiO_2 (5cM)	-0.30	0.00	3.62	1.77	4.77	0.62	0.27	0.62	-0.07
Mn-TiO_2 (6cM)	-0.30	0.00	3.60	1.65	4.79	0.72	0.34	0.72	-0.03
Ni-CH_3	-0.30	0.00	3.22	1.54	4.30	0.45	0.15	0.45	-0.15
Cu-F	-0.30	0.00	3.55	1.74	4.72	0.58	0.26	0.58	-0.07
$SrCuO_3$	-0.30	0.00	4.15	2.03	5.01	0.90	0.26	0.90	-0.37
$SrZnO_3$	-0.30	0.00	4.56	2.26	5.11	1.07	0.19	1.07	-0.69
$LaCuO_3$	-0.30	0.00	4.32	2.12	5.09	0.97	0.26	0.97	-0.46
Ni-TiO_2 (5cM)	-0.30	0.00	3.95	1.74	4.49	0.98	0.15	0.98	-0.68
$LaScO_3$	-0.30	0.00	4.20	1.53	4.58	1.45	0.30	1.45	-0.85
$LaGaO_3$	-0.30	0.00	4.29	1.66	4.72	1.40	0.30	1.40	-0.80
CaO	-0.30	0.00	4.78	2.03	4.74	1.53	0.13	1.53	-1.27
Cr-TiO_2 (5cM)	0.30	0.00	3.02	2.15	5.17	0.92	0.28	-0.36	0.92
Mn-TiO_2 (5cM)	-0.25	0.00	2.90	1.68	4.58	0.45	0.22	-0.01	0.45
Co-F	0.30	-0.48	2.57	1.34	3.91	0.11	0.05	-0.00(19)	0.11
V-TiO_2 (6cM)	-0.30	0.00	2.80	0.73	4.26	0.84	0.54	0.84	0.24
V-TiO_2 (6cM)	-0.30	0.00	2.87	0.83	4.33	0.81	0.52	0.81	0.24
Co-FGM	-0.27	0.00	2.40	0.95	3.85	0.22	0.22	0.22	0.22
$LaRuO_3$	0.30	-0.14	2.47	1.04	3.91	0.20	0.20	0.20	0.20
CoO	0.30	-0.14	2.59	1.08	4.09	0.28	0.28	0.28	0.28
Fe-TiO_2 (5cM)	-0.30	0.00	3.29	1.21	4.64	0.85	0.49	0.85	0.13
Ru-TiO_2 (6cM)	-0.30	0.00	3.24	1.13	4.66	0.88	0.54	0.88	0.20
Ru-TiO_2 (6cM)	-0.30	0.00	3.30	1.18	4.65	0.89	0.51	0.89	0.13
Ir-TiO_2 (6cM)	-0.30	0.00	3.18	1.15	4.64	0.80	0.52	0.80	0.24
Ir-TiO_2 (6cM)	-0.30	0.00	3.05	1.06	4.56	0.76	0.52	0.76	0.29
V-TiO_2 (5cM)	-0.24	0.00	2.69	0.95	4.43	0.51	0.51	0.51	0.51
Mo-TiO_2 (6cM)	-0.00(39)	0.00	2.34	0.58	4.10	0.53	0.53	0.53	0.53
Ru-TiO_2 (6cM)	-0.30	0.00	2.38	0.20	4.23	0.95	0.79	0.95	0.63
$Sr_5Na_2Ru_8O_{24}$	-0.02	0.00	2.62	0.92	4.32	0.47	0.47	0.47	0.47

LSNMR@Ru	-0.06	0.00	2.16	0.48	3.83	0.44	0.44	0.44	0.44
Mo-TiO_2 (6cM)	0.21	0.00	2.61	0.84	4.38	0.54	0.54	0.54	0.54
Cu-NH_2	0.00(28)	0.00	2.71	0.92	4.49	0.55	0.55	0.55	0.55
FeO	0.30	0.00	2.37	0.68	4.05	0.45	0.45	0.45	0.45
NiO	0.09	0.00	2.67	1.13	4.21	0.31	0.31	0.31	0.31
Ir-TiO_2 (5cM)	0.30	-0.08	2.41	0.92	3.90	0.26	0.26	0.26	0.26
Ru-FGM	0.30	-0.17	2.32	0.88	3.76	0.21	0.21	0.21	0.21
Ni-NH_2	-0.21	0.00	2.68	1.11	4.25	0.34	0.34	0.34	0.34
Rh-FGM	-0.13	0.00	2.59	1.11	4.07	0.25	0.25	0.25	0.25
Mn-FGM	0.30	-0.58	2.49	1.18	3.80	0.08	0.08	0.08	0.08
LSNMR@Mn	-0.30	0.00	2.52	0.66	3.78	0.63	0.33	0.63	0.03
Sr_2MIrO_6	0.16	0.00	2.44	0.93	3.41	0.28	0.01	0.28	-0.26
$LaCoO_3$	-0.30	-0.94	1.62	1.19	3.27	0.42	-0.19	-0.80	0.42
Cr-NH_2	0.30	-0.56	1.21	0.40	3.06	0.63	0.10	-0.43	0.63
Mn-CH_3	0.30	-0.56	1.34	0.48	3.13	0.56	0.09	-0.37	0.56
Mn-NH_2	0.30	-0.49	1.06	0.25	2.99	0.70	0.14	-0.42	0.70
$LaTiO_3$	0.30	-0.68	-1.22	-0.86	1.85	1.84	0.12	-1.59	1.84
TiO	0.30	-0.67	-1.02	-0.76	1.95	1.74	0.12	-1.49	1.74
VO	0.30	-0.57	-0.35	-0.41	2.29	1.40	0.12	-1.16	1.40
CrO	0.30	-0.33	0.83	0.16	2.87	0.82	0.12	-0.57	0.82
Mo-TiO_2 (5cM)	0.30	-1.02	1.09	0.73	3.00	0.69	-0.09	-0.87	0.69
Mo-TiO_2 (5cM)	0.30	-0.97	2.07	0.92	3.49	0.20	0.06	-0.08	0.20
Mo-TiO_2 (6cM)	0.30	-0.94	1.46	0.59	3.19	0.50	0.07	-0.36	0.50
W-TiO_2 (5cM)	0.30	-1.23	1.02	0.65	2.97	0.72	-0.07	-0.86	0.72
Mn-F	0.30	-0.68	1.73	0.76	3.33	0.36	0.05	-0.26	0.36
Mn-BH_2	0.30	-0.78	2.02	1.05	3.47	0.22	-0.02	-0.26	0.22
Fe-H	0.30	-0.72	1.79	0.93	3.35	0.34	-0.02	-0.37	0.34
Fe-F	0.30	-0.71	1.97	0.98	3.44	0.25	0.00(32)	-0.24	0.25
Fe-CH_3	0.30	-0.72	1.71	0.87	3.31	0.38	-0.01	-0.40	0.38
Fe-BH_2	0.30	-0.87	2.11	1.18	3.52	0.17	-0.06	-0.29	0.17
Co-OH	0.30	-0.67	2.26	1.18	3.59	0.10	-0.03	-0.16	0.10
Co-NH_2	0.30	-0.77	1.86	1.07	3.39	0.30	-0.07	-0.44	0.30
$SrCrO_3$	0.30	-1.07	1.52	1.06	3.22	0.47	-0.15	-0.76	0.47
$LaCrO_3$	0.30	-0.88	1.30	0.86	3.11	0.58	-0.11	-0.80	0.58
$LaMnO_3$	0.30	-0.70	1.99	0.95	3.46	0.23	0.02	-0.19	0.23
W-TiO_2 (5cM)	0.30	-0.87	0.27	0.07	2.59	1.10	0.03	-1.03	1.10
W-TiO_2 (6cM)	0.30	-0.91	0.86	0.36	2.89	0.80	0.04	-0.73	0.80
Cr-H	0.30	-0.65	1.44	0.56	3.18	0.51	0.08	-0.35	0.51
Cr-F	0.30	-0.67	1.60	0.68	3.26	0.43	0.06	-0.31	0.43
Cr-OH	0.30	-0.65	1.36	0.52	3.14	0.55	0.08	-0.39	0.55
Cr-CH_3	0.30	-0.67	1.22	0.48	3.07	0.62	0.06	-0.49	0.62
Cr-BH_2	0.30	-0.65	1.54	0.66	3.23	0.46	0.06	-0.35	0.46
Mn-H	0.30	-0.60	1.53	0.57	3.23	0.46	0.10	-0.27	0.46
Mn-OH	0.30	-0.68	1.34	0.52	3.13	0.56	0.07	-0.41	0.56
Fe-OH	0.30	-0.62	1.68	0.78	3.30	0.39	0.03	-0.33	0.39
Fe-NH_2	0.30	-0.54	1.50	0.64	3.21	0.48	0.05	-0.37	0.48
$SrVO_3$	0.30	-0.93	0.53	0.43	2.72	0.97	-0.08	-1.13	0.97
$LaVO_3$	0.30	-0.87	-0.15	0.08	2.39	1.30	-0.07	-1.45	1.30
Cr-FGM	0.30	-0.70	1.49	0.65	3.21	0.48	0.05	-0.39	0.48
ScO	0.30	0.00	-0.30	-1.22	2.28	1.41	0.52	-0.31	1.35

c) Materials with $n = 3$

Material	δ	ε	$\Delta G_{O,\delta+\varepsilon}$	$\Delta G_{OH,\delta+\varepsilon}$	$\Delta G_{OOH,\delta}$	$\eta_{OER,\delta+\varepsilon}$	$\gamma_{OOH/OH,\delta+\varepsilon}$	$\gamma_{O/OH,\delta+\varepsilon}$	$\gamma_{OOH/O,\delta+\varepsilon}$
Fe-TiO_2 (5cM)	-0.30	0.00	3.13	1.81	4.65	0.58	0.19	0.09	0.30
$SrNiO_3$	-0.30	0.00	3.25	1.88	4.63	0.65	0.15	0.14	0.15
Cr-TiO_2 (6cM)	-0.30	0.00	3.55	1.43	4.93	0.89	0.52	0.89	0.16
Cr-TiO_2 (6cM)	-0.30	0.00	3.25	1.27	4.74	0.75	0.51	0.75	0.27
Fe-TiO_2 (6cM)	-0.30	0.00	3.21	1.26	4.68	0.72	0.48	0.72	0.25

Fe-TiO_2 (6cM)	-0.30	0.00	3.38	1.26	4.76	0.89	0.52	0.89	0.16
$SrTiO_3$	-0.30	0.00	3.31	1.31	4.61	0.77	0.42	0.77	0.07
Cu-OH	-0.30	0.00	3.09	1.32	4.64	0.54	0.43	0.54	0.32
Ru-TiO_2 (5cM)	0.30	-0.35	2.75	1.36	4.14	0.16	0.16	0.16	0.16
Co-H	0.29	0.00	2.88	1.50	4.38	0.27	0.21	0.15	0.27
Co-CH_3	0.30	-0.19	2.75	1.31	4.19	0.21	0.21	0.21	0.21
$SrMnO_3$	0.30	-0.30	2.90	1.51	4.41	0.28	0.22	0.16	0.28
$SrRuO_3$	0.30	-0.07	2.86	1.41	4.32	0.22	0.22	0.22	0.22
Fe-FGM	0.30	-0.31	2.72	1.34	4.10	0.15	0.15	0.15	0.15
PtO_2	0.30	-0.06	2.95	1.51	4.46	0.28	0.25	0.21	0.28
Ir-TiO_2 (5cM)	-0.27	0.00	2.93	1.49	4.42	0.26	0.23	0.21	0.26
Ni-BH_2	-0.30	0.00	3.25	1.63	4.49	0.40	0.20	0.39	0.01
Cu-H	-0.30	0.00	3.52	1.77	4.77	0.54	0.27	0.52	0.02
Ni-TiO_2 (6cM)	-0.30	0.00	3.67	1.74	5.01	0.70	0.41	0.70	0.12
Ni-H	-0.30	0.00	3.06	1.52	4.55	0.31	0.28	0.31	0.26
Cu-CH_3	-0.30	0.00	3.16	1.54	4.59	0.39	0.30	0.39	0.20
$BaNiO_3$	-0.30	0.00	3.28	1.57	4.69	0.48	0.33	0.48	0.18
$BaNiO_{3\text{-d2}}$	-0.30	0.00	3.34	1.48	4.85	0.63	0.46	0.63	0.28
V-TiO_2 (5cM)	0.30	-0.17	2.95	1.68	4.62	0.45	0.24	0.04	0.45
Ir-FGM	0.30	0.00	3.02	1.55	4.57	0.32	0.28	0.24	0.32
Ir-TiO_2 (6cM)	0.16	0.00	3.05	1.30	4.80	0.52	0.52	0.52	0.52
Ni-F	-0.18	0.00	3.08	1.68	4.76	0.45	0.31	0.17	0.45
$SrCoO_3$	-0.05	0.00	2.91	1.49	4.40	0.26	0.22	0.19	0.26
$BaNiO_{3\text{-d1}}$	-0.24	0.00	3.07	1.69	4.76	0.46	0.30	0.14	0.46
$BaNiO_2$	-0.27	0.00	2.93	1.58	4.51	0.35	0.23	0.11	0.35
Cr-TiO_2 (5cM)	-0.07	0.00	3.31	1.55	5.07	0.53	0.53	0.53	0.53
Ru-TiO_2 (5cM)	-0.12	0.00	3.31	1.62	5.00	0.46	0.46	0.46	0.46
Ni-OH	-0.03	0.00	3.15	1.55	4.76	0.37	0.37	0.37	0.37
$LaNiO_3$	-0.02	0.00	3.06	1.52	4.60	0.31	0.31	0.31	0.31
$Sr_{7/8}Na_{1/8}RuO_3$	-0.17	0.00	2.83	1.39	4.26	0.20	0.20	0.20	0.20
$Sr_{6/8}Na_{2/8}RuO_3$	-0.14	0.00	3.04	1.45	4.62	0.35	0.35	0.35	0.35
$Sr_7Ru_8O_{24}$	-0.08	0.00	3.22	1.50	4.94	0.49	0.49	0.49	0.49
$Sr_6Na_1Ru_8O_{24}$	-0.24	0.00	2.97	1.38	4.55	0.35	0.35	0.35	0.35
Cr-TiO_2 (6cM)	0.09	0.00	3.09	1.33	4.85	0.53	0.53	0.53	0.53
Co-BH_2	0.29	0.00	3.05	1.53	4.58	0.30	0.30	0.30	0.30
$SrFeO_3$	0.02	0.00	3.02	1.69	4.71	0.46	0.28	0.10	0.46
$LaFeO_3$	0.15	0.00	3.05	1.40	4.70	0.42	0.42	0.42	0.42
RuO_2	0.13	0.00	2.98	1.48	4.48	0.27	0.27	0.27	0.27
MnO_2	0.00	0.00	3.20	1.84	5.04	0.61	0.37	0.13	0.61
Sr_2MIrO_6	0.30	-0.30	2.08	0.71	3.50	0.19	0.17	0.14	0.19
Sr_2MIrO_6	0.30	-0.18	2.11	0.60	3.62	0.28	0.28	0.28	0.28
Sr_2MIrO_6	0.07	0.00	1.77	0.07	3.47	0.47	0.47	0.47	0.47
W-TiO_2 (6cM)	0.30	-0.39	2.07	0.52	3.62	0.32	0.32	0.32	0.32
W-TiO_2 (6cM)	0.30	-0.56	1.76	0.36	3.34	0.35	0.26	0.17	0.35
MnO	0.30	-0.49	2.03	0.72	3.47	0.22	0.15	0.08	0.22
V-TiO_2 (6cM)	-0.30	0.00	2.16	0.09	3.67	0.84	0.56	0.84	0.29
Nb-TiO_2 (6cM)	-0.30	0.00	2.10	-0.00(47)	3.50	0.87	0.52	0.87	0.18
Nb-TiO_2 (5cM)	-0.15	0.00	1.84	0.09	3.59	0.52	0.52	0.52	0.52
Nb-TiO_2 (6cM)	-0.30	0.00	1.79	-0.24	3.29	0.80	0.54	0.80	0.28
Ta-TiO_2 (6cM)	-0.30	0.00	1.57	-0.65	3.07	0.99	0.63	0.99	0.28
Ta-TiO_2 (6cM)	-0.30	0.00	1.95	-0.17	3.34	0.89	0.53	0.89	0.17
Nb-TiO_2 (6cM)	-0.30	0.00	1.74	-0.19	3.31	0.70	0.52	0.70	0.35
Ta-TiO_2 (5cM)	-0.07	0.00	1.36	-0.58	2.98	0.71	0.55	0.71	0.38
Ta-TiO_2 (5cM)	-0.11	0.00	1.64	-0.13	3.41	0.54	0.54	0.54	0.54
Ta-TiO_2 (6cM)	-0.30	0.00	1.74	-0.22	3.30	0.73	0.53	0.73	0.34
Nb-TiO_2 (5cM)	0.27	0.00	1.92	0.18	3.66	0.51	0.51	0.51	0.51
IrO_2	0.22	0.00	2.09	0.51	3.67	0.35	0.35	0.35	0.35

Table S5. $\delta\varepsilon$ optimized values of ΔG_O , ΔG_{OH}, ΔG_{OOH} (in eV), η_{OER} (in V), $\gamma_{OOH/OH}$, $\gamma_{OH/O}$ and $\gamma_{OOH/O}$ (in V) classified according to the number of electrochemical steps over 1.23 eV (n).

a) Materials with $n = 2$

Material	δ	ε	$\Delta G_{O,\delta\varepsilon}$	$\Delta G_{OH,\delta\varepsilon}$	$\Delta G_{OOH,\delta e}$	$\eta_{OER,\delta\varepsilon}$	$\gamma_{OOH/OH,\delta e}$	$\gamma_{O/OH,\delta e}$	$\gamma_{OOH/O,\delta e}$
Ni-BH_2	-0.70	-0.40	2.46	1.23	3.69	0.00(25)	0.00(05)	- 0.00(15)	0.00(25)
Fe-TiO_2 (5cM)	-0.60	-0.93	2.52	1.50	3.42	0.27	-0.27	-0.21	-0.33
Ni-FGM	-0.86	-0.42	2.44	1.28	3.64	0.05	-0.05	-0.08	-0.03
Mn-TiO_2 (5cM)	-0.51	-0.81	2.36	1.41	3.51	0.18	-0.18	-0.28	-0.09
$BaNiO_{3-d1}$	-0.55	-0.90	2.43	1.38	3.54	0.15	-0.15	-0.17	-0.12
MnO_2	-0.45	-1.07	2.31	1.39	3.53	0.16	-0.16	-0.32	-0.01
Sr_2ScIrO_6	0.47	0.00	2.43	0.88	3.97	0.32	0.32	0.31	0.32
Sr_2NiIrO_6	0.16	0.00	2.44	0.93	3.41	0.28	0.01	0.28	-0.26
$Sr_6Na_1Ru_8O_{24}$	-0.50	-0.69	2.44	1.12	3.60	0.09	0.01	0.09	-0.06
$SrMnO_3$	0.01	-0.80	2.33	1.23	3.62	0.07	-0.03	-0.13	0.07
$SrZnO_3$	-1.42	-0.36	2.32	1.14	3.62	0.07	0.01	-0.04	0.07
Fe-OH	0.57	-0.62	2.23	1.05	3.57	0.12	0.03	-0.06	0.12

b) Materials with $n = 3$

Material	δ	ε	$\Delta G_{O,\delta\varepsilon}$	$\Delta G_{OH,\delta\varepsilon}$	$\Delta G_{OOH,\delta e}$	$\eta_{OER,\delta\varepsilon}$	$\gamma_{OOH/OH,\delta e}$	$\gamma_{O/OH,\delta e}$	$\gamma_{OOH/O,\delta e}$
Fe-F	0.56	-0.69	2.48	1.23	3.72	0.02	0.02	0.02	0.02
Fe-TiO_2 (5cM)	-0.91	-0.53	2.50	1.25	3.67	0.02	-0.02	0.01	-0.06
Cu-CH_3	-0.58	-0.73	2.59	1.25	3.58	0.11	-0.07	0.11	-0.24
$BaNiO_3$	-0.61	-0.86	2.66	1.26	3.52	0.17	-0.10	0.17	-0.38
Sr_2FeIrO_6	0.32	-0.44	2.16	0.62	3.39	0.30	0.15	0.30	0.00(08)
Sr_2CoIrO_6	0.01	-0.13	1.65	0.01	3.28	0.41	0.41	0.41	0.40
TiO_2	-1.18	-0.32	2.24	0.89	3.58	0.11	0.11	0.11	0.11
V-TiO_2 (6cM)	-0.65	-0.13	1.47	-0.26	3.19	0.50	0.50	0.50	0.50
V-TiO_2 (6cM)	-0.80	-0.42	1.81	0.23	3.34	0.35	0.33	0.35	0.31
V-TiO_2 (6cM)	-0.81	-0.44	1.85	0.32	3.38	0.31	0.30	0.31	0.30
Nb-TiO_2 (6cM)	-0.64	0.00	1.41	-0.35	3.16	0.53	0.52	0.53	0.52
Co-FGM	-0.36	-0.19	2.21	0.86	3.57	0.12	0.12	0.12	0.12
LSNMR@Mn	-0.63	-0.07	1.87	0.33	3.39	0.30	0.30	0.30	0.29
Mo-TiO_2 (5cM)	0.44	-0.97	2.34	1.05	3.63	0.06	0.06	0.06	0.06
Mo-TiO_2 (6cM)	0.73	-0.94	2.32	1.01	3.62	0.07	0.07	0.07	0.07
Ru-TiO_2 (5cM)	0.15	-0.66	2.44	1.20	3.68	0.01	0.01	0.01	0.01
Mn-F	0.61	-0.68	2.36	1.07	3.64	0.05	0.05	0.05	0.05
Co-CH_3	0.12	-0.54	2.38	1.13	3.65	0.04	0.03	0.03	0.04
$SrRuO_3$	0.09	-0.49	2.44	1.20	3.68	0.01	0.01	0.01	0.01
$LaMnO_3$	0.51	-0.70	2.42	1.17	3.67	0.02	0.02	0.02	0.02
$LaRuO_3$	0.20	-0.35	2.27	0.94	3.59	0.10	0.10	0.10	0.10
CoO	0.13	-0.48	2.25	0.91	3.58	0.11	0.11	0.11	0.11
Mn-FGM	0.24	-0.67	2.36	1.12	3.64	0.05	0.03	0.01	0.05
Fe-FGM	0.16	-0.58	2.44	1.20	3.68	0.01	0.01	0.01	0.01
Cr-TiO_2 (6cM)	-1.02	-0.70	2.11	0.71	3.52	0.17	0.17	0.17	0.17
Cr-TiO_2 (6cM)	-0.87	-0.66	2.10	0.69	3.51	0.18	0.18	0.18	0.18
Mn-TiO_2 (5cM)	-0.90	-0.50	2.42	1.16	3.67	0.02	0.02	0.02	0.02
Mn-TiO_2 (6cM)	-0.94	-0.55	2.32	1.01	3.61	0.08	0.07	0.08	0.06
Mn-TiO_2 (6cM)	-1.13	-0.37	2.23	0.88	3.57	0.12	0.11	0.12	0.11
Mn-TiO_2 (6cM)	-1.12	-0.41	2.25	0.89	3.57	0.12	0.11	0.12	0.09
Fe-TiO_2 (6cM)	-0.85	-0.62	2.12	0.71	3.51	0.18	0.17	0.18	0.17
Fe-TiO_2 (6cM)	-0.98	-0.61	2.01	0.57	3.47	0.22	0.22	0.21	0.22
Fe-TiO_2 (5cM)	-0.93	-0.54	2.02	0.57	3.47	0.22	0.22	0.22	0.22
Ru-TiO_2 (6cM)	-0.93	-0.59	1.97	0.49	3.44	0.25	0.24	0.25	0.24

Ru-TiO_2 (6cM)	-0.96	-0.54	1.99	0.52	3.45	0.24	0.24	0.24	0.24
Ir-TiO_2 (6cM)	-0.88	-0.59	2.02	0.56	3.47	0.22	0.22	0.22	0.22
Ir-TiO_2 (6cM)	-0.83	-0.58	2.00	0.53	3.45	0.24	0.23	0.24	0.23
Ni-TiO_2 (5cM)	-1.16	-0.06	2.22	0.87	3.57	0.12	0.12	0.12	0.12
Ni-TiO_2 (6cM)	-0.95	-0.72	2.36	1.08	3.64	0.05	0.05	0.05	0.05
Ni-TiO_2 (6cM)	-1.15	-0.34	2.26	0.93	3.59	0.10	0.10	0.10	0.10
Ni-TiO_2 (6cM)	-1.09	-0.38	2.28	0.95	3.59	0.10	0.09	0.10	0.08
Ni-H	-0.60	-0.56	2.45	1.22	3.69	0.00(43)	0.00(31)	0.00(20)	0.00(43)
Ni-CH_3	-0.71	-0.23	2.39	1.13	3.66	0.03	0.03	0.03	0.03
Cu-F	-0.86	-0.48	2.43	1.18	3.67	0.02	0.02	0.02	0.02
Cu-BH_2	-1.34	-0.23	1.94	0.44	3.42	0.27	0.26	0.27	0.25
$SrScO_3$	-1.50	-0.13	2.23	0.89	3.58	0.11	0.11	0.11	0.11
$SrTiO_3$	-0.90	-0.49	2.11	0.71	3.52	0.17	0.17	0.17	0.17
$SrCuO_3$	-1.17	-0.49	2.42	1.16	3.66	0.03	0.02	0.03	0.01
$SrGeO_3$	-1.23	-0.54	1.98	0.51	3.45	0.24	0.24	0.24	0.24
$LaScO_3$	-1.44	-0.07	1.93	0.39	3.38	0.31	0.26	0.31	0.21
$LaCuO_3$	-1.25	-0.47	2.42	1.17	3.67	0.02	0.02	0.02	0.02
$LaZnO_3$	-1.41	-0.23	2.36	1.07	3.63	0.06	0.05	0.06	0.04
$LaGaO_3$	-1.46	-0.12	1.98	0.50	3.45	0.24	0.24	0.24	0.24
CaO	-1.59	0.00	2.20	0.73	3.45	0.24	0.13	0.24	0.02
$BaNiO_{3-d2}$	-0.83	-0.72	2.27	0.95	3.59	0.10	0.09	0.10	0.09
LSNMR@Ni	-0.82	-0.10	2.08	0.65	3.50	0.19	0.19	0.19	0.19
V-TiO_2 (5cM)	-0.55	-0.63	2.06	0.63	3.49	0.20	0.20	0.20	0.20
Nb-TiO_2 (5cM)	-0.26	-0.21	1.63	-0.02	3.27	0.42	0.42	0.42	0.42
Mo-TiO_2 (6cM)	-0.10	-0.62	1.99	0.53	3.46	0.23	0.23	0.23	0.23
Mo-TiO_2 (6cM)	-0.24	-0.47	1.87	0.35	3.40	0.29	0.29	0.29	0.29
W-TiO_2 (6cM)	0.24	-0.52	1.94	0.45	3.43	0.26	0.26	0.26	0.26
Ru-TiO_2 (6cM)	-0.75	-0.60	1.48	-0.25	3.19	0.50	0.49	0.50	0.48
Ir-TiO_2 (6cM)	-0.25	-0.82	2.23	0.89	3.58	0.11	0.11	0.11	0.11
Cu-NH_2	-0.34	-0.68	2.03	0.58	3.47	0.22	0.22	0.22	0.22
FeO	0.10	-0.41	1.96	0.48	3.44	0.25	0.25	0.25	0.25
NiO	-0.12	-0.41	2.25	0.92	3.59	0.10	0.10	0.10	0.10
$Sr_5Na_2Ru_8O_{24}$	-0.29	-0.55	2.07	0.65	3.49	0.20	0.19	0.20	0.19
LSNMR@Ru	-0.20	-0.30	1.87	0.34	3.39	0.30	0.30	0.30	0.30
Nb-TiO_2 (6cM)	-0.50	0.00	1.38	-0.45	3.09	0.60	0.54	0.60	0.48
Ta-TiO_2 (6cM)	-0.49	0.00	1.19	-0.84	2.89	0.80	0.63	0.80	0.46
Ta-TiO_2 (6cM)	-0.57	0.00	1.40	-0.45	3.07	0.62	0.53	0.62	0.44
W-TiO_2 (5cM)	1.36	-0.87	2.39	1.13	3.66	0.03	0.03	0.03	0.03
W-TiO_2 (6cM)	1.06	-0.91	2.39	1.12	3.65	0.04	0.04	0.04	0.04
W-TiO_2 (6cM)	0.40	-0.59	1.96	0.46	3.41	0.28	0.25	0.28	0.22
Ir-TiO_2 (5cM)	0.18	-0.32	2.17	0.80	3.55	0.15	0.15	0.15	0.15
Cr-H	0.73	-0.65	2.30	0.99	3.61	0.08	0.08	0.08	0.08
Cr-F	0.67	-0.67	2.34	1.05	3.63	0.06	0.06	0.06	0.06
Cr-OH	0.77	-0.65	2.30	0.99	3.61	0.08	0.08	0.08	0.08
Cr-CH_3	0.86	-0.57	2.33	1.04	3.63	0.06	0.06	0.06	0.06
Cr-BH_2	0.70	-0.65	2.35	1.06	3.63	0.06	0.06	0.06	0.06
Cr-NH_2	0.83	-0.56	2.26	0.93	3.59	0.10	0.10	0.10	0.10
Mn-H	0.67	-0.60	2.26	0.94	3.59	0.10	0.10	0.10	0.10
Mn-OH	0.78	-0.68	2.31	1.01	3.62	0.07	0.07	0.07	0.07
Mn-CH_3	0.76	-0.56	2.27	0.95	3.60	0.09	0.09	0.09	0.09
Mn-NH_2	0.86	-0.49	2.18	0.81	3.55	0.14	0.14	0.14	0.14
Fe-NH_2	0.73	-0.54	2.35	1.07	3.64	0.05	0.05	0.05	0.05
$LaTiO_3$	2.02	-0.68	2.21	0.86	3.57	0.12	0.12	0.12	0.12
ScO	1.16	0.00	1.42	-0.35	3.14	0.55	0.52	0.55	0.49
TiO	1.91	-0.67	2.21	0.86	3.57	0.12	0.12	0.12	0.12
VO	1.58	-0.57	2.22	0.87	3.57	0.12	0.12	0.12	0.12
CrO	0.99	-0.33	2.21	0.86	3.57	0.12	0.12	0.12	0.12
MnO	0.38	-0.50	2.18	0.80	3.54	0.15	0.14	0.15	0.13
Cr-FGM	0.74	-0.70	2.36	1.09	3.64	0.05	0.05	0.05	0.05
Ru-FGM	0.25	-0.35	2.22	0.83	3.53	0.16	0.12	0.16	0.09

Cr-TiO_2 (5cM)	-0.55	-0.96	2.35	1.07	3.64	0.05	0.05	0.05	0.05
Cr-TiO_2 (6cM)	-0.35	-0.85	2.20	0.88	3.56	0.13	0.11	0.09	0.13
Ru-TiO_2 (5cM)	-0.56	-0.89	2.42	1.17	3.67	0.02	0.02	0.02	0.02
Ni-OH	-0.39	-0.72	2.43	1.19	3.68	0.01	0.01	0.01	0.01
Ni-NH_2	-0.44	-0.45	2.23	0.88	3.57	0.12	0.12	0.12	0.12
Cu-OH	-0.73	-0.63	2.24	0.90	3.58	0.11	0.11	0.11	0.11
$LaFeO_3$	-0.21	-0.72	2.34	1.04	3.63	0.06	0.06	0.06	0.06
$LaNiO_3$	-0.32	-0.61	2.45	1.22	3.69	0.00(47)	0.00(27)	0.00(47)	0.00(08)
Rh-FGM	-0.26	-0.37	2.33	0.98	3.57	0.12	0.07	0.12	0.02
$Sr_{7/8}Na_{1/8}RuO_3$	-0.36	-0.39	2.44	1.20	3.68	0.01	0.01	0.01	0.01
$Sr_{6/8}Na_{2/8}RuO_3$	-0.46	-0.64	2.39	1.13	3.66	0.03	0.03	0.03	0.03
Sr7Ru8O_{24}	-0.51	-0.87	2.35	1.07	3.63	0.06	0.05	0.06	0.05
RuO_2	-0.13	-0.53	2.45	1.22	3.68	0.01	0.00(50)	0.01	0.00(49)
Nb-TiO_2 (5cM)	0.15	-0.24	1.68	0.06	3.30	0.39	0.39	0.39	0.39
Nb-TiO_2 (6cM)	-0.46	0.00	1.41	-0.36	3.15	0.54	0.52	0.54	0.51
Ta-TiO_2 (5cM)	-0.07	0.00	1.36	-0.58	2.98	0.71	0.55	0.71	0.38
Ta-TiO_2 (5cM)	-0.18	-0.14	1.51	-0.20	3.21	0.48	0.48	0.48	0.48
Ta-TiO_2 (6cM)	-0.47	0.00	1.39	-0.40	3.13	0.56	0.53	0.56	0.51
IrO_2	0.17	-0.23	1.99	0.46	3.39	0.30	0.24	0.30	0.17
Mo-TiO_2 (5cM)	0.90	-1.02	2.28	1.32	3.60	0.09	-0.09	-0.27	0.09
W-TiO_2 (5cM)	0.95	-1.23	2.32	1.30	3.62	0.07	-0.07	-0.20	0.07
Mn-BH_2	0.50	-0.78	2.42	1.25	3.67	0.02	-0.02	-0.06	0.02
Fe-H	0.62	-0.72	2.42	1.25	3.67	0.02	-0.02	-0.05	0.02
Fe-CH_3	0.67	-0.72	2.44	1.24	3.68	0.01	-0.01	-0.03	0.01
Fe-BH_2	0.42	-0.88	2.35	1.30	3.62	0.07	-0.07	-0.18	0.05
Co-H	0.03	-0.48	2.37	1.24	3.64	0.05	-0.03	-0.10	0.05
Co-F	0.19	-0.64	2.36	1.23	3.64	0.05	-0.03	-0.11	0.05
Co-OH	0.36	-0.67	2.37	1.24	3.65	0.04	-0.03	-0.10	0.04
Co-NH_2	0.53	-0.77	2.32	1.30	3.62	0.07	-0.07	-0.21	0.07
$SrCrO_3$	0.63	-1.09	2.18	1.38	3.54	0.15	-0.15	-0.43	0.12
$LaCrO_3$	0.77	-0.88	2.24	1.34	3.58	0.11	-0.11	-0.32	0.11
PtO_2	0.04	-0.59	2.43	1.25	3.67	0.02	-0.02	-0.05	0.02
Ir-TiO_2 (5cM)	-0.51	-0.49	2.43	1.24	3.68	0.01	-0.01	-0.04	0.01
Ni-TiO_2 (5cM)	-1.10	-0.39	2.39	1.28	3.64	0.05	-0.05	-0.13	0.02
Cu-H	-0.84	-0.55	2.45	1.24	3.68	0.01	-0.01	-0.02	0.00(18)
$SrNiO_3$	-0.82	-0.55	2.21	1.36	3.56	0.13	-0.13	-0.38	0.12
CuO	-0.76	-0.33	2.42	1.25	3.67	0.02	-0.02	-0.06	0.02
Cu-FGM	-1.06	-0.39	2.41	1.26	3.66	0.03	-0.03	-0.08	0.03
Pd-FGM	-1.19	-0.30	2.28	1.32	3.60	0.09	-0.09	-0.27	0.09
Ag-FGM	-1.22	-0.29	2.32	1.30	3.62	0.07	-0.07	-0.21	0.06
Pt-FGM	-1.14	-0.34	2.35	1.29	3.63	0.06	-0.06	-0.17	0.06
Au-FGM	-1.29	-0.23	2.26	1.33	3.59	0.10	-0.10	-0.30	0.10
V-TiO_2 (5cM)	-0.04	-0.86	2.26	1.33	3.59	0.10	-0.10	-0.30	0.10
Cr-TiO_2 (5cM)	-0.30	-1.20	1.82	1.55	3.37	0.32	-0.32	-0.96	0.32
$LaCoO_3$	-0.07	-0.94	2.08	1.42	3.50	0.19	-0.19	-0.57	0.19
Ir-FGM	0.00	-0.60	2.42	1.25	3.67	0.02	-0.02	-0.05	0.02
$SrVO_3$	1.18	-0.93	2.29	1.31	3.61	0.08	-0.08	-0.25	0.08
$LaVO_3$	1.53	-0.87	2.31	1.30	3.62	0.07	-0.07	-0.22	0.07
Co-BH_2	-0.01	-0.59	2.46	1.23	3.69	0.00(02)	-0.00(02)	-0.00(05)	0.00(02)
Ni-F	-0.56	-0.76	2.32	1.30	3.62	0.07	-0.07	-0.21	0.07
$SrFeO_3$	-0.35	-0.74	2.28	1.32	3.60	0.09	-0.09	-0.27	0.09
$SrCoO_3$	-0.29	-0.49	2.43	1.25	3.67	0.02	-0.02	-0.06	0.01
$BaNiO_2$	-0.56	-0.59	2.34	1.29	3.63	0.06	-0.06	-0.18	0.06

The values of δ and the resulting η_{OER} are generally different for δ, $\delta + \varepsilon$ and $\delta\varepsilon$ optimizations. For example: $SrMnO_3$ has $\delta = 0.45\ eV$ and $\eta_{OER} = 0.43\ V$ upon δ

optimization; $\delta = 0.30\ eV$ and $\eta_{OER} = 0.28\ V$ upon $\delta + \varepsilon$ optimization, and $\delta = 0.01\ eV$ and $\eta_{OER} = 0.07\ V$ upon $\delta\varepsilon$ optimization. The differences arise because δ is free in δ optimization, constrained to the range of ±0.3 eV in $\delta + \varepsilon$ optimization, and coupled to ε in $\delta\varepsilon$ optimization.

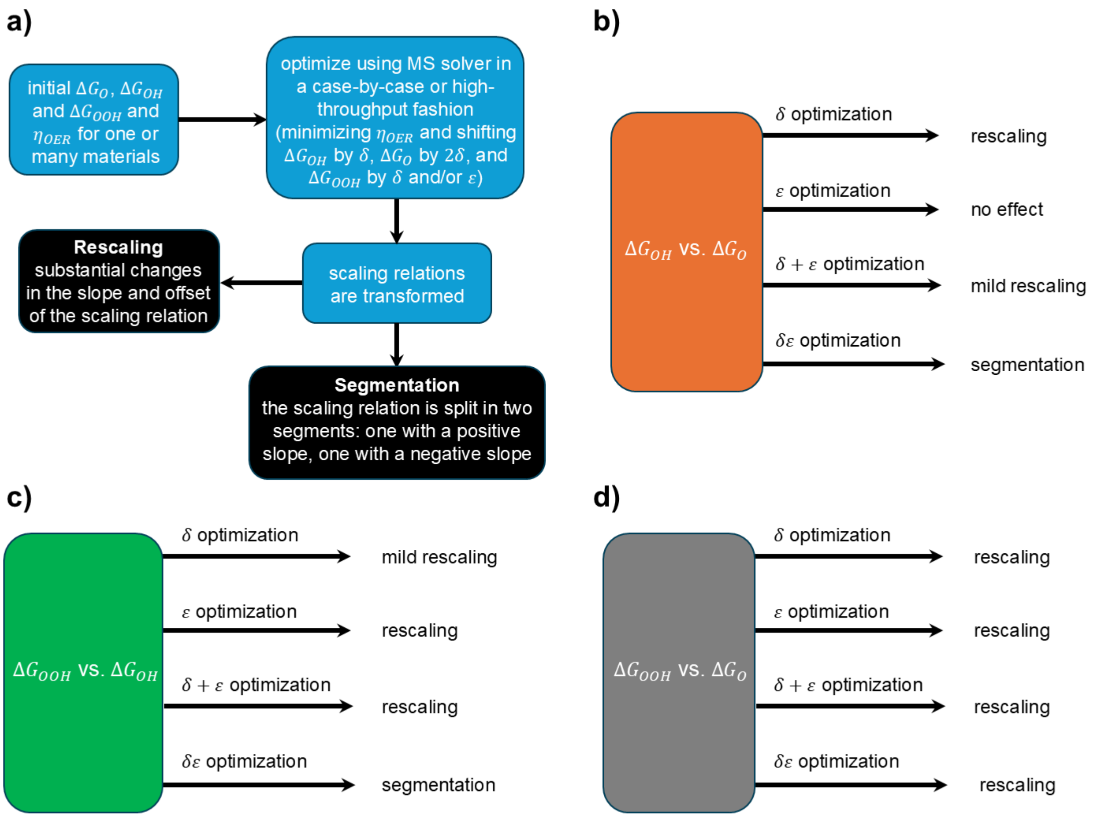

Figure S1. a) Summarized workflow to observe rescaling and segmentation. The transformations observed as a result of the optimizations are listed for b) ΔG_{OH} vs. ΔG_O, c) ΔG_{OOH} vs. ΔG_{OH}, and d) ΔG_{OOH} vs. ΔG_O.

S3. Transformation of scaling relations upon $\delta - \varepsilon$ optimization

Equation 16 ($m^*(1 + \Delta\epsilon'/\Delta\Delta G'_{OH}) = m$) in the main text is general and predicts that statistical deviations from a linear fit cause changes in the slopes of scaling relations upon optimization. In the following, we will make a specific analysis for *OH vs *O under delta optimization and copy the results for the other representative scaling relations for conciseness.

According to the formalism in the main text, for δ optimization we have: $\Delta G'_{OH} = \Delta G_{OH} + \delta$, where ΔG_{OH} is the initial *OH adsorption energy and $\Delta G'_{OH}$ is the final one. Because of scaling relations, $\Delta G'_{O} = \Delta G_{O} + \delta/m$, where m is the initial slope. When the initial scaling relation is transformed from $\Delta G_{OH} = m\Delta G_{O} + a$ into $\Delta G'_{OH} = m_\delta \Delta G'_{O} + b$, an original data point $(\Delta G_{O_1}, \Delta G_{OH_1})$ for material 1 is transformed into $(\Delta G'_{O_1}, \Delta G'_{OH_1})$. We note that, as explained in section 3.1, there are statistical deviations in the data (ϵ). Hence, the scaling relations for materials 1 and 2 are:

$$\Delta G'_{OH_1} = m_\delta \Delta G'_{O_1} + b + \epsilon_1 \tag{S4}$$

$$\Delta G'_{OH_2} = m_\delta \Delta G'_{O_2} + b + \epsilon_2 \tag{S5}$$

Applying the above definitions, we have:

$$\Delta G_{OH_1} + \delta_1 = m_\delta(\Delta G_{O_1} + \delta_1/m) + b + \epsilon_1 \tag{S6}$$

$$\Delta G_{OH_2} + \delta_2 = m_\delta(\Delta G_{O_2} + \delta_2/m) + b + \epsilon_2 \tag{S7}$$

Combining equations S6-S7 and solving for m_δ we have:

$$m_\delta = m\left(1 - \frac{\epsilon_2 - \epsilon_1}{\Delta G_{OH_2} - \Delta G_{OH_1} + \delta_2 - \delta_1}\right) = m\left(1 - \frac{\Delta\epsilon}{\Delta\Delta G_{OH} + \Delta\delta}\right) \tag{S8}$$

It is worth noting that the new slope depends on the *OH adsorption energies, the errors and the values of δ. For ε optimization, where $\Delta G'_{OOH} = \Delta G_{OOH} + \varepsilon$ and $\Delta G'_{OH} = \Delta G_{OH}$, it is possible to show that the new slope (m_ε) for *OOH vs *OH is:

$$m_\varepsilon = m\left(1 + \frac{\Delta\varepsilon - \Delta\epsilon}{\Delta\Delta G_{OOH}}\right) \tag{S9}$$

In this case, the new slope depends on the *OOH adsorption energies, the errors and the values of ε. For $\delta\varepsilon$ optimization, where $\Delta G'_{OOH} = \Delta G_{OOH} + \delta + \varepsilon$ and $\Delta G'_{OH} = \Delta G_{OH} + \delta$, one can show that the new slope ($m_{\delta\varepsilon}$) for *OOH vs. *O is:

$$m_{\delta\varepsilon} = m\left(1 + \frac{\Delta\varepsilon - \Delta\epsilon}{\Delta\Delta G_{OOH} + \Delta\delta}\right) \quad \text{(S10)}$$

In this final example, the new slope depends on the *OOH adsorption energies, the errors and the values of both δ and ε. In summary, equation 16 in the main text predicts the transformation of scaling relations and equations S8-S10 show that the slopes will change as a function of the adsorption energies, the errors and the parameters of delta-epsilon optimization.

S4. Origin of the transformations after delta-epsilon optimizations

The hinge point of the segments is the ideal catalyst ($\Delta G_{OH,ideal} = 1.23\ eV$, $\Delta G_{O,ideal} = 2.46\ eV$), such that negative segments appear for $\Delta G_{OH,opt} > 1.23\ eV$, and the positive segments for $\Delta G_{OH,opt} < 1.23\ eV$. Materials on the negative segment of ΔG_{OH} vs. ΔG_O are also such that $\Delta G_{O,opt} < 2.46\ eV$. This is because the ideal catalyst belongs to the negative segment and the slope is $m_{neg} = (\Delta G_{OH} - \Delta G_{OH,ideal})/(\Delta G_O - \Delta G_{O,ideal})$. Hence, the numerator in the negative segment is typically positive ($\Delta G_{OH} - \Delta G_{OH,ideal} > 0$) and the denominator negative ($\Delta G_O - \Delta G_{O,ideal} < 0$).

Combining the above expressions, it follows that a material will be on the negative segment when $\Delta G_{O,opt} - 2\Delta G_{OH,opt} < 0$. Because δ is a scaling-based parameter, this energetic difference is the same before and after all optimizations ($\Delta G_{O,initial} - 2\Delta G_{OH,initial} = \Delta G_{O,opt} - 2\Delta G_{OH,opt}$). For example, let us consider the case of $SrNiO_3$, which has $\Delta G_O = 3.85\ eV$ and $\Delta G_{OH} = 2.18\ eV$. After $\delta\varepsilon$ optimization with $\delta = -0.82\ eV$ and $\varepsilon = -0.55\ eV$, this perovskite displays $\Delta G_O = 2.21\ eV$ and $\Delta G_{OH} = 1.36\ eV$. Because $\Delta G_O - 2\Delta G_{OH} = 3.85 - 2 \cdot 2.18 = 2.21 - 1.36 \cdot 2 = -0.51\ eV \leq 0$, this catalyst is on the negative segment of the scaling relation. As shown in Figure S2, the materials on the negative segment correspond to the upper part of the original scaling relation of ΔG_{OH} vs. ΔG_O (in general, all the points on or above the line $\Delta G_{OH} = 0.5\ \Delta G_O$).

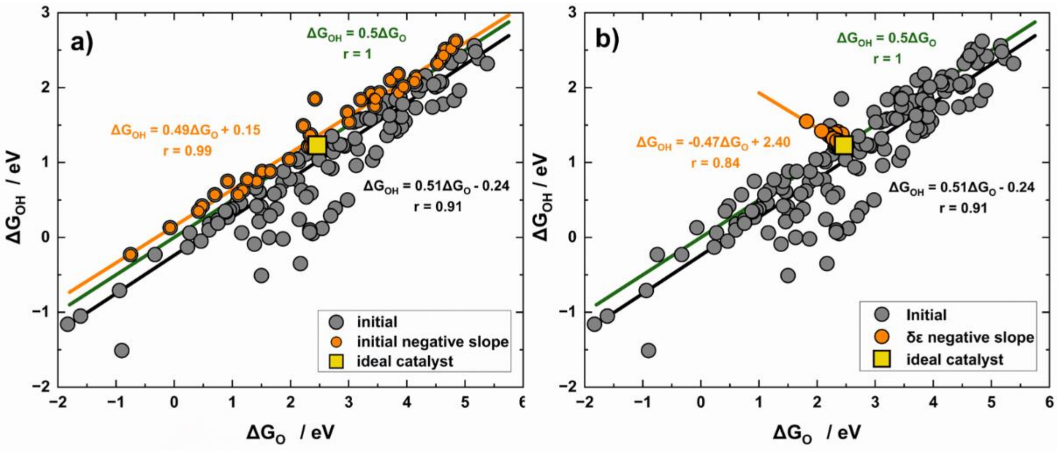

Figure S2. Scaling relationship of ΔG_{OH} vs. ΔG_O for (a) the initial dataset (gray) highlighting the subset of materials that upon $\delta\varepsilon$ optimization form the negative-slope segment (orange), and (b) the initial dataset (gray) together with the corresponding $\delta\varepsilon$ -optimized values of the materials belonging to the negative-slope segment (orange). The ideal catalyst ($\eta_{OER} = 0$ and $n = 4$) is shown as a yellow square. The line corresponding to $\Delta G_{OH} = 0.5\Delta G_O$ is provided for comparison.

The change in slope upon δ optimization originates from the intrinsic scattering of the data points around the initial scaling relation. Because δ optimization is applied to a dataset that deviates from perfect linearity, that is the correlation coefficient is not 1.00, the differences between the individual data points and the linear fit lead to a modified slope and intercept. To illustrate this effect, we analyze in the following the ΔG_{OH} *vs.* ΔG_O scaling relation for the family of single-atom-catalysts (SACs) in Tables S1-S2. Figure S3a shows the initial and δ-optimized scaling relations for Co- and Mn-based FGM materials, where δ is adjusted by enforcing the original relation in Table S6. In this two-material example, the δ-optimized points converge to the same position, which belongs to the original scaling relation. When a third material (Fe-FGM) is included in the trend (Figure S3b), the rescaling is then clear, such that both the slope and intercept increase noticeably upon δ-optimization. Figure S3c further illustrates that, upon incorporating the full SAC family, the deviations in both slope and intercept accumulate, consistent with

the broader spread of the dataset. Again, the slopes and intercepts of the initial and δ-optimized scaling lines are reported in Table S6.

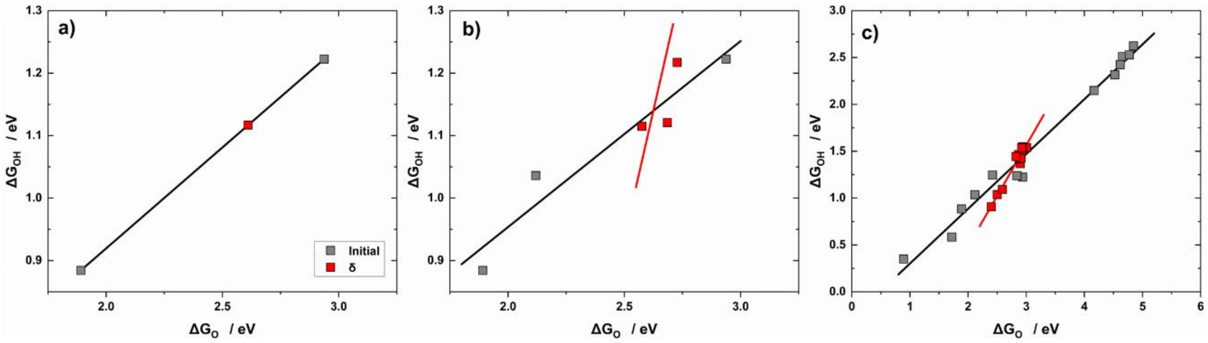

Figure S3. Effect of introducing δ optimization on the ΔG_{OH} *vs.* ΔG_O scaling relation. Initial and δ-optimized scaling lines shown for (a) two materials, (b) three materials, and (c) the full SAC family using data from Table S1.

Table S6. Scaling relations of ΔG_{OH} *vs.* ΔG_O ($\Delta G_{OH} = m\Delta G_O + \xi$) derived from initial and optimized data for 2 materials only, 3 materials only, and the full SAC family in Table S1. Reported values include the correlation coefficient (r).

Number of materials	Data set	m	ξ (eV)	r
2 materials (Co, Mn FGM)	Initial	0.32	0.27	1.00
2 materials (Co, Mn FGM)	δ-optimized	−	−	1.00
3 materials (Co, Mn, Fe FGM)	Initial	0.30 ± 0.08	0.36 ± 0.18	0.97
3 materials (Co, Mn, Fe FGM)	δ-optimized	1.63 ± 0.05	-3.15 ± 0.13	0.99
Full SAC family	Initial	0.58 ± 0.02	-0.28 ± 0.08	0.99
Full SAC family	δ-optimized	1.08 ± 0.08	-1.67 ± 0.21	0.97

S5. Dependence of the slopes on n

Dissecting Figures 1 and 3 in the main text, we observe that an overall scaling relation has trends within it as a function of n. Scaling relations as a function of n are listed in Tables S7 (ΔG_{OH} *vs.* ΔG_O) and S8 (ΔG_{OOH} *vs.* ΔG_O). Importantly, we observe that materials with $n = 3$, which typically display high OER activity, tend to have slopes close to 1, while the expected slopes are 0.5.

Table S7. Scaling relations of ΔG_{OH} *vs.* ΔG_O ($\Delta G_{OH} = m\Delta G_O + \xi$) derived from initial and optimized data as a function of n. Reported values include the correlation coefficient (r).

	n	m	$\xi\ (eV)$	r
Initial	2	0.52 ± 0.02	-0.29 ± 0.06	0.94
Initial	3	1.00 ± 0.07	-1.68 ± 0.22	0.94
δ	2	1.39 ± 0.13	-2.58 ± 0.35	0.81
δ	3	1.12 ± 0.02	-1.91 ± 0.07	0.98
$\delta + \varepsilon$	2	0.49 ± 0.02	-0.15 ± 0.06	0.92
$\delta + \varepsilon$	3	1.08 ± 0.05	-1.87 ± 0.15	0.93

Table S8. Scaling relations of ΔG_{OOH} *vs.* ΔG_O ($\Delta G_{OOH} = m\Delta G_O + \xi$) derived from initial and optimized data as a function of n. Reported values include the correlation coefficient (r).

	n	m	$\xi\ (eV)$	r
Initial	2	0.45 ± 0.01	3.06 ± 0.05	0.96
Initial	3	0.86 ± 0.05	1.94 ± 0.16	0.95
δ	2	1.12 ± 0.11	1.33 ± 0.28	0.81
δ	3	1.00 ± 0.02	1.61 ± 0.06	0.98
ε	3	0.93 ± 0.04	1.68 ± 0.12	0.97
$\delta + \varepsilon$	2	0.60 ± 0.02	2.43 ± 0.05	0.97
$\delta + \varepsilon$	3	0.96 ± 0.03	1.66 ± 0.08	0.97

S6. δ optimization using least-squares and theoretical slopes

δ is a scaling-based parameter and was applied to *O, *OH, and *OOH. The slopes of scaling relations are rooted in electron-counting arguments.[2,3] While the least-squares slopes of ΔG_{OH} vs. ΔG_O and ΔG_{OOH} vs. ΔG_O are not strictly 0.5, the observed slopes of 0.51 and 0.45 (Table 3) are close enough.[4–9] The larger deviations for ΔG_{OOH} vs. ΔG_{OH} (0.82 vs. 1.00) occur due to pronounced covalence effects in compounds of late transition metals.[10] As shown in Figure S4, one can carry out delta-epsilon optimizations using the least-squares initial slopes instead of the theoretical ones and the results are consistent with those in the main text. In Figure S4 we observe a rescaling of all scaling relations upon δ optimization with slopes and intercepts similar to those obtained using the theoretical slopes. The slopes and intercepts for each scaling relation obtained using the theoretical and actual slopes, are reported in Table S9.

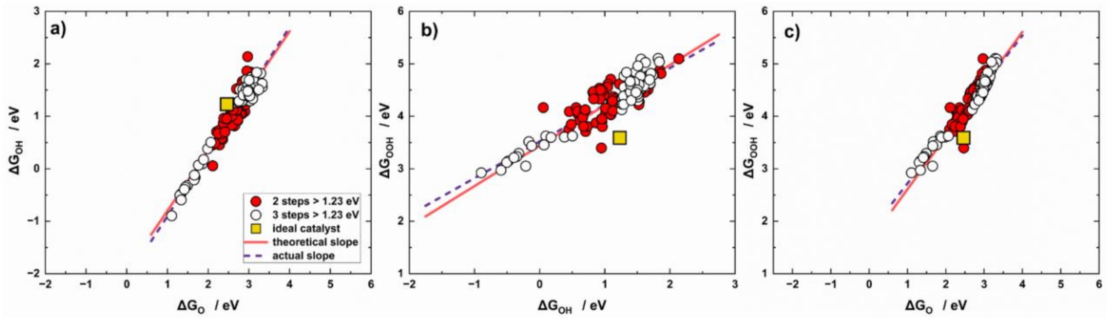

Figure S4. Scaling relations upon δ optimization made with the initial least-squares slopes for (a) ΔG_{OH} vs. ΔG_O, (b) ΔG_{OOH} vs. ΔG_{OH}, and (c) ΔG_{OOH} vs. ΔG_O. The data are classified depending on n: red ($n = 2$), and white ($n = 3$). The ideal catalyst ($\eta_{OER} = 0$ and $n = 4$) is shown as a yellow square. Solid lines indicate theoretical scaling relations and dashed lines the least-squares slopes.

Table S9. Final slopes and intercepts of the linear fits upon δ optimization, using the theoretical (Th) and least-squares (LS) slopes. Th/LS: theoretical/least-squares values of the slopes.

Scaling relation	Slope used	m	$\xi\ (eV)$	r
$\Delta G_{OH}\ vs.\ \Delta G_O$	0.50 (Th)	1.14 ± 0.03	-1.94 ± 0.08	0.95
$\Delta G_{OH}\ vs.\ \Delta G_O$	0.51 (LS)	1.20 ± 0.03	-2.10 ± 0.08	0.96
$\Delta G_{OOH}\ vs.\ \Delta G_{OH}$	1.00 (Th)	0.77 ± 0.03	3.44 ± 0.04	0.88
$\Delta G_{OOH}\ vs.\ \Delta G_{OH}$	0.82 (LS)	0.70 ± 0.03	3.52 ± 0.04	0.86
$\Delta G_{OOH}\ vs.\ \Delta G_O$	0.50 (Th)	1.00 ± 0.02	1.61 ± 0.07	0.96
$\Delta G_{OOH}\ vs.\ \Delta G_O$	0.45 (LS)	0.94 ± 0.02	1.78 ± 0.07	0.95

S7. ΔG_{OOH} vs. ΔG_{O} upon $\delta\varepsilon$ optimization excluding large values of δ and ε

The nature of delta-epsilon optimizations is deterministic. That means that η_{OER} of a given material with known ΔG_O, ΔG_{OH}, ΔG_{OOH} will be minimized by the exact same values of δ and ε if the optimizations are carried out numerous times. In addition, delta-epsilon optimizations can be carried out individually or in a high-throughput fashion, and the results are identical in both cases because each material has its own values of δ and ε, so material 1 has δ_1, material 2 has δ_2, etcetera, and δ_1, δ_2… are independent from each other and the same holds true for ε.

Scaling relations are said in our manuscript to be statistical in the sense that there are deviations between the individual data points and the overall linear fit. Those deviations are responsible for the rescaling and segmentation of the lines, as shown in equation 16. Nevertheless, each material is deterministically optimized, such that the global scaling behavior is the sum of all individual contributions. In Figure S5b we have excluded the data points for which the magnitudes of δ and/or ε are larger than the adsorption energies (~33% of the data) and the initial trends persist.

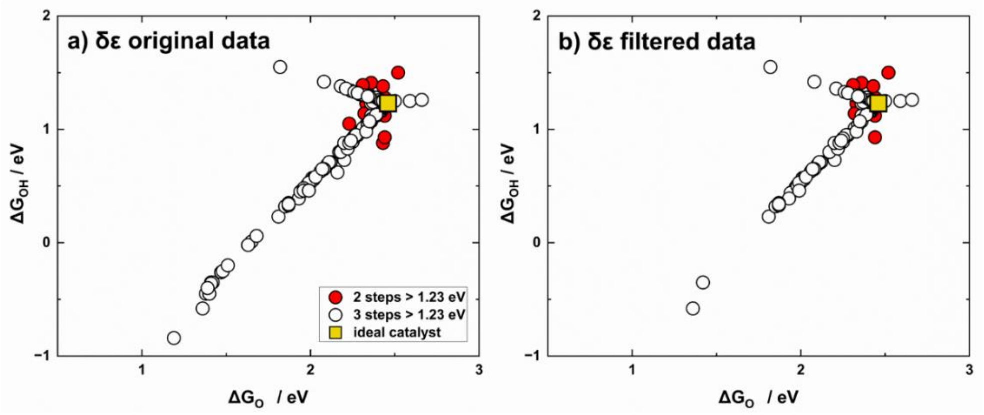

Figure S5. ΔG_{OH} vs. ΔG_O upon simultaneous $\delta\varepsilon$ optimization: (a) original data, and (b) filtered data excluding large optimization parameters (δ and/or ε), where data points for which the magnitudes of δ and/or ε exceed the adsorption energies (~33% of the dataset) have been removed. Segmentation of the original scaling relation is observed in both cases. The ideal catalyst ($\eta_{OER} = 0$ and $n = 4$) is shown as a yellow square.

The derivation of the ΔG_{OOH} *vs* ΔG_O scaling relation upon $\delta\varepsilon$ optimization from the segmented ΔG_{OH} *vs* ΔG_O and ΔG_{OOH} *vs* ΔG_{OH} relations (see Tables 1-3 in the main text) requires combining the segments α and β, associated to materials with $\Delta G_{OH} <$ 1.23 eV (72% of data entries) and $\Delta G_{OH} >$ 1.23 eV (28% of data entries), respectively. The process is shown in Table S10.

Table S10. Construction of the α and β segments as combinations of segments $(\delta\varepsilon)_1$ and $(\delta\varepsilon)_2$ listed in Tables 1 and 2 of the main text.

Segment	Condition	segment	Scaling relation	m	ξ (eV)	Weight (%)
α	$\Delta G_{OH} < 1.23$ eV	$(\delta\varepsilon)_1$	ΔG_{OH} *vs.* ΔG_O	1.53 ± 0.02	-2.54 ± 0.04	
		$(\delta\varepsilon)_1$	ΔG_{OOH} *vs.* ΔG_{OH}	0.35 ± 0.01	3.26 ± 0.01	
			ΔG_{OOH} *vs.* ΔG_O	0.54 ± 0.02	2.36 ± 0.03	72
β	$\Delta G_{OH} > 1.23$ eV	$(\delta\varepsilon)_2$	ΔG_{OH} *vs.* ΔG_O	-0.34 ± 0.06	2.10 ± 0.15	
		$(\delta\varepsilon)_2$	ΔG_{OOH} *vs.* ΔG_{OH}	-0.94 ± 0.06	4.83 ± 0.07	
			ΔG_{OOH} *vs.* ΔG_O	0.32 ± 0.06	2.86 ± 0.20	28
$0.72\alpha + 0.28\beta$			ΔG_{OOH} *vs.* ΔG_O	0.48 ± 0.02	2.50 ± 0.06	100
$\delta\varepsilon$			ΔG_{OOH} *vs.* ΔG_O	0.51 ± 0.02	2.42 ± 0.03	100

Table S10 shows how the α and β segments in the main text are constructed by pairing segments $(\delta\varepsilon)_1$ and $(\delta\varepsilon)_2$, from the ΔG_O *vs.* ΔG_{OH} and ΔG_{OH} *vs* ΔG_{OOH} scaling relations. The α segment is obtained by combining the two $(\delta\varepsilon)_1$ lines, while the β segment is obtained analogously from $(\delta\varepsilon)_2$. These combinations yield the slope (m) and intercept (ξ) reported in the main text, which are obtained as the weighted contributions of the α (72%) and β (28%) segments. The scaling relation obtained this way is close to the one obtained directly from the optimized data (δε) in Table 3, and the resulting ΔG_{OOH} *vs*. ΔG_O is not segmented.

S8. Analysis of $\gamma_{O/OH}$ and $\gamma_{OOH/O}$

In the main text, the breaking of the ΔG_{OOH} *vs.* ΔG_{OH} scaling relation was analyzed using $\gamma_{OOH/O}$. Here, we extend the analysis to ΔG_O *vs.* ΔG_{OH} and ΔG_{OOH} *vs.* ΔG_O. The parameter $\gamma_{O/OH}$, defined according to equation S11, is numerically equivalent to $U_2 = \Delta G_2/e^-$.

$$\gamma_{O/OH} = (\Delta G_O - \Delta G_{OH} - 1.23)/1e^- \qquad \text{(S11)}$$

The ideal catalyst exhibits $\gamma_{O/OH} = 0$. As shown in Figure S6a, the initial dataset displays a V shape, where materials with $n = 3$ are around the apex and the remaining materials fall on both sides. In general, $\gamma_{O/OH}$ decreases upon applying δ, with some materials falling close to the ideal value. This is also observed after applying ε. However, as this parameter does not affect *OH and *O, the V shape is retained while shifting to lower η_{OER} for materials with $\varepsilon \neq 0$. $\delta + \varepsilon$ optimization yields a similar trend compared to ε optimization. Finally, upon $\delta\varepsilon$ optimization an inverted volcano is observed again, wherein most materials are near the apex and there are different slopes on each side. This result is consistent with the segmentation observed in the analysis of the scaling relations.

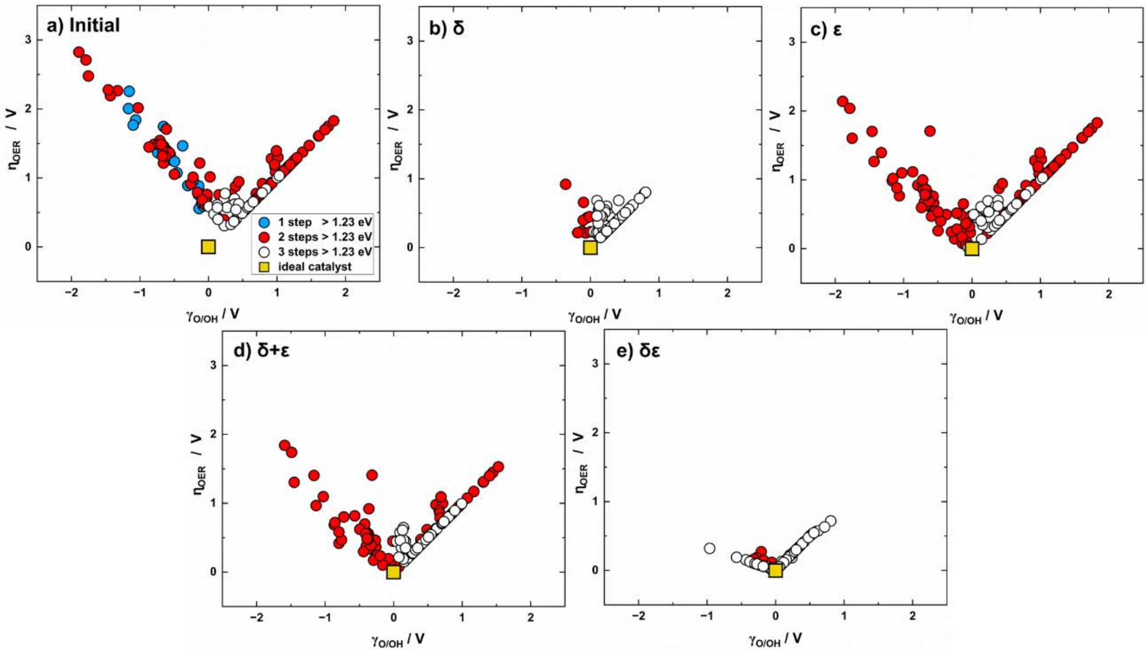

Figure S6. η_{OER} as a function of the materials departures from the ideal *O *vs* *OH energetic separation ($\gamma_{O/OH}$). The data are classified depending on the number of electrochemical steps above 1.23 eV at 0 V *vs* RHE: blue ($n = 1$), red ($n = 2$), and white ($n = 3$).

In turn, $\gamma_{OOH/O}$ is defined according to equation S12 and numerically corresponds to $U_3 = \Delta G_3/e^-$. In the initial dataset (Figure S7a), a V shape is observed and materials with $n = 3$ are close to the apex, in analogy to the behavior seen for *OH *vs.* *O. Figure S3b shows that δ optimization shifts the materials toward the right side of the curve and places them close to the ideal region. Since ε only modifies materials for which step 3 is the potential limiting step (PLS), its application results in a shift of the right side of the curve toward lower η_{OER}, see Figure S7c. A similar trend is obtained when the sequential optimization is applied (Figure S7d). In Figure S7e, the simultaneous optimization ($\delta\varepsilon$) drives all materials close to the ideal value.

$$\gamma_{OOH/O} = (\Delta G_{OOH} - \Delta G_O - 1.23)/1e^- \quad \text{(S12)}$$

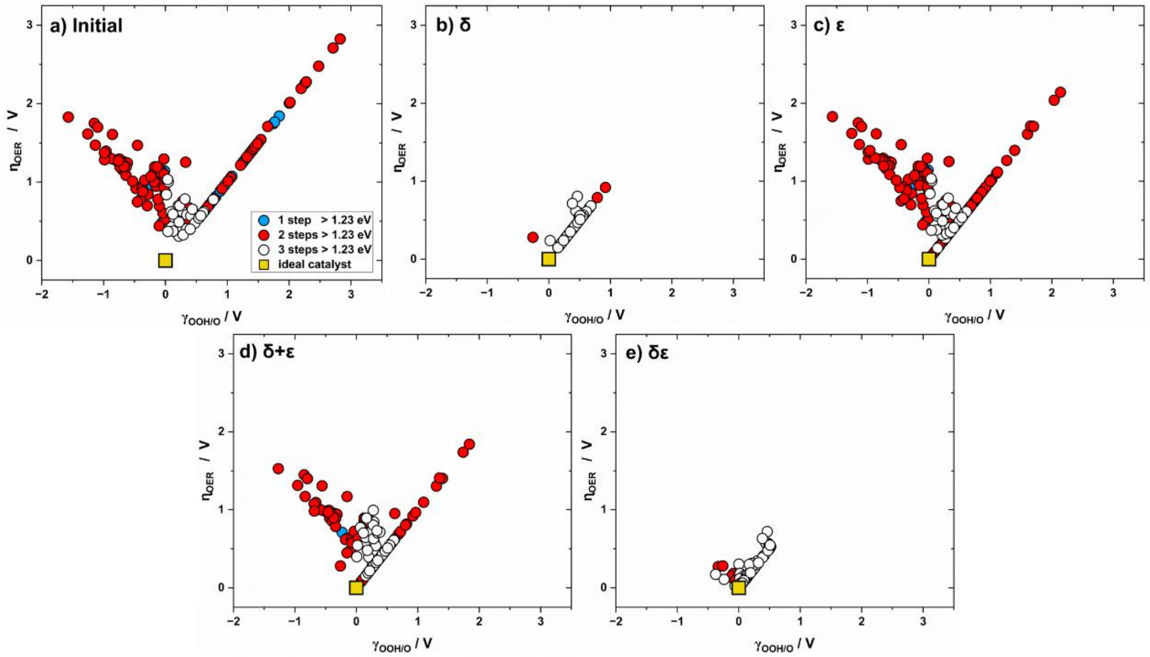

Figure S7. η_{OER} as a function of the materials departures from the ideal *OOH *vs* *O energetic separation ($\gamma_{OOH/O}$). The data are classified depending on the number of electrochemical steps above 1.23 eV at 0 V *vs* RHE: blue ($n = 1$), red ($n = 2$), and white ($n = 3$).

References

(1) Nørskov, J. K.; Rossmeisl, J.; Logadottir, A.; Lindqvist, L.; Kitchin, J. R.; Bligaard, T.; Jónsson, H. Origin of the Overpotential for Oxygen Reduction at a Fuel-Cell Cathode. *J. Phys. Chem. B* **2004**, *108* (46), 17886–17892. https://doi.org/10.1021/jp047349j.

(2)Abild-Pedersen, F.; Greeley, J.; Studt, F.; Rossmeisl, J.; Munter, T. R.; Moses, P. G.; Skúlason, E.; Bligaard, T.; Nørskov, J. K. Scaling Properties of Adsorption Energies for Hydrogen-Containing Molecules on Transition-Metal Surfaces. *Phys. Rev. Lett.* **2007**, *99* (1), 016105. https://doi.org/10.1103/PhysRevLett.99.016105.

(3) Calle-Vallejo, F.; Martínez, J. I.; García-Lastra, J. M.; Rossmeisl, J.; Koper, M. T. M. Physical and Chemical Nature of the Scaling Relations between Adsorption Energies of Atoms on Metal Surfaces. *Phys. Rev. Lett.* **2012**, *108* (11), 116103. https://doi.org/10.1103/PhysRevLett.108.116103.

(4) Man, I. C.; Su, H. Y.; Calle-Vallejo, F.; Hansen, H. A.; Martínez, J. I.; Inoglu, N. G.; Kitchin, J.; Jaramillo, T. F.; Nørskov, J. K.; Rossmeisl, J. Universality in Oxygen Evolution Electrocatalysis on Oxide Surfaces. *ChemCatChem* **2011**, *3* (7), 1159–1165. https://doi.org/10.1002/cctc.201000397.

(5) Rossmeisl, J.; Logadottir, A.; Nørskov, J. K. Electrolysis of Water on (Oxidized) Metal Surfaces. *Chem. Phys.* **2005**, *319* (1–3), 178–184. https://doi.org/10.1016/j.chemphys.2005.05.038.

(6) Li, J. Oxygen Evolution Reaction in Energy Conversion and Storage: Design Strategies Under and Beyond the Energy Scaling Relationship. *Nano-Micro Lett.* **2022**, *14* (1), 1–32. https://doi.org/10.1007/s40820-022-00857-x.

(7) Vojvodic, A.; Nørskov, J. K. New Design Paradigm for Heterogeneous Catalysts. *Natl. Sci. Rev.* **2015**, *2*, 140–143.

(8) Masa, J.; Schuhmann, W. Breaking Scaling Relations in Electrocatalysis. *J. Solid State Electrochem.* **2020**, *24* (9), 2181–2182. https://doi.org/10.1007/s10008-020-04757-1.

(9) Exner, K. S. Why the Breaking of the OOH versus OH Scaling Relation Might Cause Decreased Electrocatalytic Activity. *Chem Catal.* **2021**, *1* (2), 258–271. https://doi.org/10.1016/j.checat.2021.06.011.

(10) Calle-Vallejo, F.; Krabbe, A.; García-Lastra, J. M. How Covalence Breaks Adsorption-Energy Scaling Relations and Solvation Restores Them. *Chem. Sci.* **2016**, *8* (1), 124–130. https://doi.org/10.1039/C6SC02123A.
